# Perioperative Risk Factors for Permanent Pacemaker Implantation After Transcatheter Aortic Valve Replacement: A Systematic Review and Meta-Analysis

**DOI:** 10.31083/RCM39299

**Published:** 2025-10-23

**Authors:** Xi Peng, Nan Chen, Peng Li, Fang-Hui Zhu, Ming Li, Xiao-Han Zhao, Hui-Ping Zhang

**Affiliations:** ^1^Department of Cardiology, Beijing Hospital, National Center of Gerontology, Institute of Geriatric Medicine, Chinese Academy of Medical Sciences, 100730 Beijing, China; ^2^Arrhythmia Center, Fuwai Hospital, National Center for Cardiovascular Diseases, Chinese Academy of Medical Sciences, Peking Union Medical College, 100730 Beijing, China; ^3^The Key Laboratory of Geriatrics, Beijing Institute of Geriatrics, Institute of Geriatric Medicine, Chinese Academy of Medical Sciences, Beijing Hospital/National Center of Gerontology of National Health Commission, 100730 Beijing, China

**Keywords:** aortic stenosis, transcathether aortic valve replacement, cardiac conduction abnormalities, permanent pacemaker implantation, perioperative risk factors

## Abstract

**Background:**

Transcatheter aortic valve replacement (TAVR) has become the preferred treatment for severe aortic stenosis, particularly in patients at high surgical risk. Conduction block requiring permanent pacemaker (PPM) implantation remains a common complication post-TAVR. This systematic review and meta-analysis aimed to clarify perioperative (≤30-day) predictors of PPM implantation.

**Methods:**

A systematic search was performed using the PubMed, Web of Science, and Embase databases to gather all relevant studies examining the relationship between TAVR and pacemaker implantation outcomes within 30 days of the procedure. Pooled odds ratios (ORs) with 95% confidence intervals (CIs) were calculated using a random-effects model.

**Results:**

A total of 82 studies comprising 124,808 patients were included. The overall incidence of PPM implantation within 30 days post-TAVR was 17.5%. Key baseline risk factors included right bundle branch block (RBBB) (OR, 5.48; 95% CI, 4.52–6.64) and first-degree atrioventricular block (AVB) (OR, 2.30; 95% CI, 1.82–2.90). Baseline left bundle branch block (LBBB), mitral annular calcification, and male sex were not significantly associated with PPM implantation. A longer membranous septum (MS) length was associated with a reduced risk (OR, 0.78; 95% CI, 0.66–0.93). Additionally, procedural risk factors included greater implant depth (OR, 1.20; 95% CI, 1.13–1.28), the use of self-expanding valves (OR, 2.59; 95% CI, 2.06–3.27), and balloon predilation (OR, 1.37; 95% CI, 1.10–1.71). The cusp overlap technique (COT) significantly reduced PPM risk (OR, 0.45; 95% CI, 0.35–0.58). Furthermore, a greater difference between MS length and implantation depth (ΔMSID) was inversely correlated with PPM implantation risk (OR, 1.36; 95% CI, 1.22–1.50), and post-TAVR new-onset LBBB was a strong predictor of PPM implantation (OR, 2.26; 95% CI, 1.66–3.07).

**Conclusions:**

This meta-analysis identified key perioperative predictors of PPM implantation following TAVR. RBBB, first-degree AVB, increased implant depth, self-expanding valves, and predilation all have been shown to increase PPM risk, whereas COT and lower ΔMSID are protective factors.

**The PROSPERO Registration:**

CRD42023438228, URL: https://www.crd.york.ac.uk/PROSPERO/view/CRD42023438228.

## 1. Introduction 

Transcatheter aortic valve replacement (TAVR) is increasingly used to treat severe aortic stenosis [1]. TAVR has become the preferred treatment option, particularly in patients who are ineligible for surgery or approximately 6.8% of patients receiving balloon-expandable valves and 23.1% of those with self-expanding systems required permanent pacemaker (PPM) within 30 days, the latter carrying a 3.4-fold higher risk [2, 3]. The occurrence of PPM following TAVR is associated with prolonged hospitalization, increased mortality, and higher rates of heart failure readmission, emphasizing the critical need for improved risk stratification, particularly among patients at high surgical risk [4]. Additionally, its use is gradually being extended to include patients at intermediate and low risks [2]. Despite procedural refinements and new generation devices have reduced complications such as vascular injury and paravalvular leak, conduction disturbances necessitating PPM implantation remained a critical concern.

The anatomical vulnerability of the His-Purkinje system to mechanical compression during valve deployment largely accounted for PPM risk [5]. A shorter membranous septum length (<3.5 mm) and deeper implantation depths amplify injury likelihood, while innovative techniques like the cusp overlap technique (COT) could potentially reduce this risk by optimizing valve positioning [6, 7]. Baseline conduction abnormalities made the situation more complicated: right bundle branch block (RBBB) increased PPM odds by 5.5-fold, and first-degree atrioventricular block (AVB) doubled the probability [6]. However, existing studies are restricted by factors such as inconsistent variable definitions (e.g., “implant depth”), inconsistent sex-based risk reporting, and a paucity of data on emerging protective strategies like COT [7].

Although several meta-analyses have been conducted on this topic, most of the existing literature has primarily focused on predictors without specifying a clear timeframe, often mixing short-term and long-term factors. This lack of distinction makes it difficult to identify perioperative predictors that specifically influence early PPM implantation risk. In contrast, our study systematically analyze perioperative risk factors specifically within the 30-day window. Therefore, this systematic review and meta-analysis aims to clarify perioperative (≤30-day) predictors of PPM implantation. By synthesizing evidence on anatomical, procedural, and post-interventional factors, we hope to provide a framework for personalized risk assessment and procedural planning, thereby addressing gaps left by prior fragmented analyses.

## 2. Materials and Methods

This review was conducted in accordance with the Preferred Reporting Items for Systematic Reviews and Meta-Analyses (PRISMA) standards, based on a systematic review and quality assessment of meta-analyses. This study has been registered with the PROSPERO International prospective register of systematic reviews (CRD42023438228). The ethical approval was waived.

### 2.1 Search Strategy and Information Sources

A systematic search was executed across the PubMed, Web of Science, and Embase databases to gather all relevant studies examining the relationship between TAVR and permanent pacemaker implantation outcomes. The search keywords were as follows: (“Transcatheter Aortic Valve Replacement” OR “Transcatheter Aortic Valve Implantation” OR “Transcatheter Aortic Valve Insertion” OR “TAVR” OR “TAVI”) AND (“pacemaker implantation” OR “permanent pacemaker” OR “pacemaker”) AND (“postoperative complications” OR “prognosis” OR “outcome” OR “risk factors” OR “predictors”). The search covered the literature published up to January 2024. Additionally, we also manually searched reference lists of included articles and relevant reviews, and scanned preprint servers (medRxiv, ResearchSquare) for unpublished studies meeting eligibility criteria. All retrieved records were managed in Endnote software, with deduplication algorithms applied before screening.

### 2.2 Inclusion and Exclusion Criteria

We included original studies that met the following criteria: (1) Population: Patients undergoing TAVR. (2) Outcome: Studies explicitly reporting of PPM implantation rates within 30 days post-procedure. (3) Design: Randomized controlled trials (RCTs) or observational studies (retrospective cohorts, Newcastle-Ottawa Scale (NOS) scores ≥7) with multivariable adjustment for confounders. (4) Risk Analysis: Examination of at least one predefined risk factor (e.g., anatomical, procedural, or electrophysiological variables) associated with 30-day PPM implantation.

Studies were excluded if they (1) involved non-human subjects or focused on basic science mechanisms; (2) lacked clear exclusion criteria for patients with preexisting pacemakers—a critical safeguard against selection bias; (3) compared TAVR with surgical valve replacement or evaluated valve brands without analyzing PPM risk factors; or (4) used aggregated public registry data, which may include duplicate individual patient records from primary studies. To minimize heterogeneity, we also excluded non-English publications and studies reporting outcomes beyond 30 days, as late conduction disturbances often reflected distinct pathophysiological mechanisms.

### 2.3 Data Collection Process

Two investigators independently performed a dual-phase screening process: initial title/abstract review followed by full-text assessment to determine study eligibility. Data extraction was restricted to articles meeting predefined quality thresholds. From eligible studies, we systematically extracted the following variables: (1) Study identifiers (first author, publication year); (2) Design (prospective/retrospective cohort, RCT); (3) Cohort characteristics (sample size, age, sex distribution, STS score); (4) Quantitative outcomes (number of pacemaker implantations within 30 days post-TAVR).

All extractions were conducted independently by two reviewers using standardized electronic forms. Discrepancies in screening decisions or data interpretation were resolved through iterative discussion, with unresolved cases adjudicated by a third senior investigator. Inter-rater agreement was quantified using Cohen’s κ coefficient (κ = 0.91 for full-text eligibility).

### 2.4 Quality Assessment

Two independent investigators evaluated methodological quality using standardized criteria. Cohort studies were assessed with the NOS, scoring selection bias (e.g., cohort representativeness), comparability (adjustment for age/comorbidities), and outcome validity (follow-up adequacy) on a 9-point scale; studies scoring <7 were excluded.

Randomized trials were appraised via the Cochrane Risk of Bias Tool (RoB 2.0, version 1, August 2019; Cochrane Methods Group, London, UK), examining randomization integrity, intervention adherence, missing data handling, outcome measurement consistency, and selective reporting-trials with ≥3 high-risk domains were excluded. Disagreements (initially 12% of assessments) were resolved through consensus discussions, with unresolved cases finalized by a third investigators.

### 2.5 Statistical Analysis

Categorical variables were summarized as counts and proportions (%), while continuous variables were reported as means with standard deviations (SD) or medians with interquartile ranges (IQR) based on distribution normality. We employed a random-effects model (DerSimonian-Laird estimator) to pool adjusted odds ratios (ORs) and 95% confidence intervals (CIs), prioritizing this approach to account for anticipated clinical and methodological heterogeneity across studies. Between-study heterogeneity was quantified using Cochran’s Q test (significance threshold: *p* < 0.10) and the *I*^2^ statistic, with *I*^2^ values interpreted as follows: ≤25% (low), 25–50% (moderate), >50% (high).

To assess small-study effects and publication bias, we generated funnel plots complemented by Egger’s regression test for asymmetry (*p* < 0.05 indicating significance). Prespecified sensitivity analyses excluded studies contributing disproportionately to heterogeneity (influence analysis) and those with NOS scores <8. All analyses were conducted using R software (version 4.3.1, metafor and dmetar packages, R Foundation for Statistical Computing, Vienna, Austria), with two-tailed *p* < 0.05 defining statistical significance.

## 3. Results

### 3.1 Study Selection

**Fig. 1.**
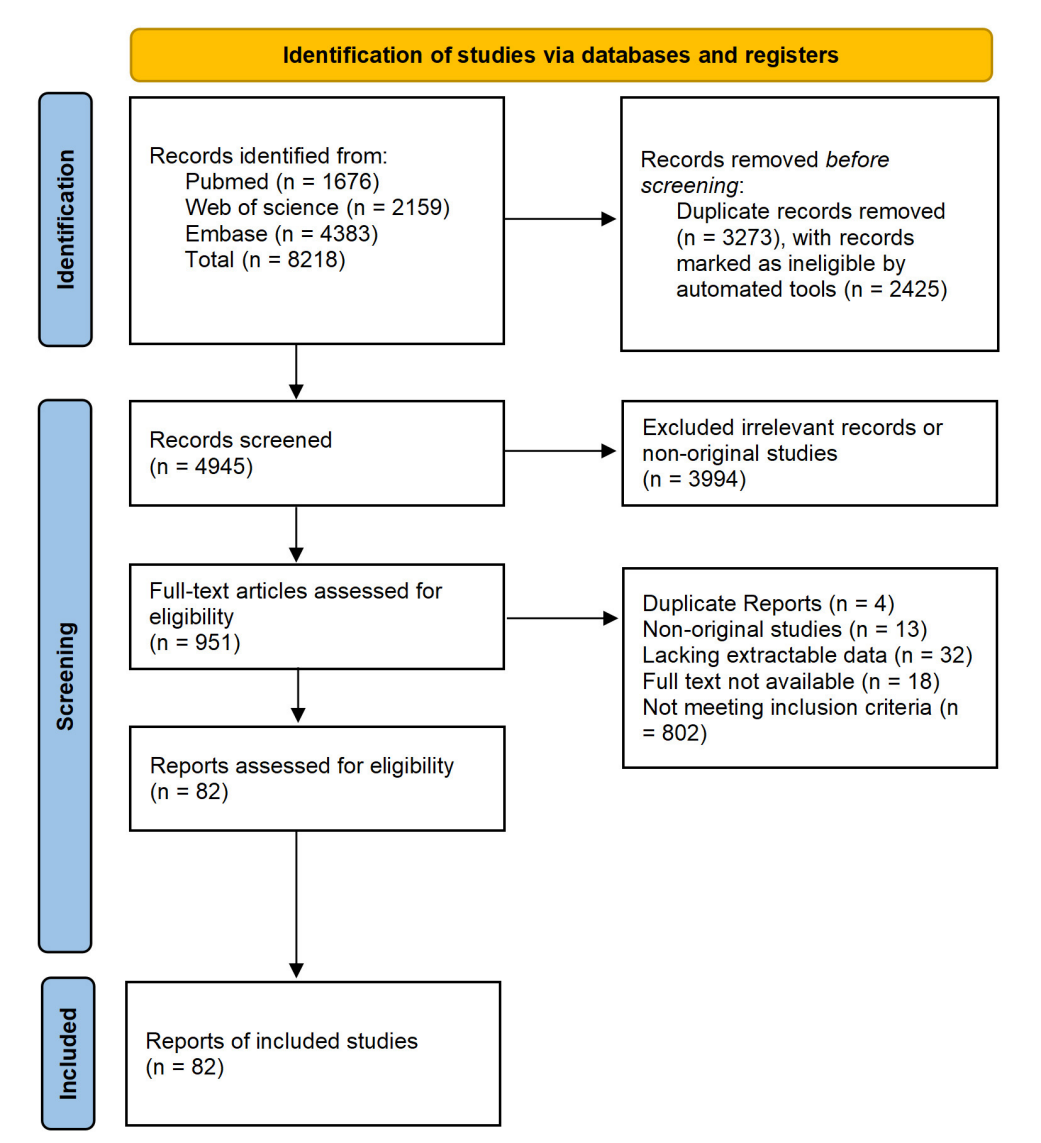
**PRISMA flow diagram for the study selection process**. PRISMA, Preferred Reporting Items for Systematic Reviews and Meta-Analyses.

Using the aforementioned search strategy, a preliminary identification of 8218 articles was performed. After removing duplicates, 4945 unique articles remained. Following an assessment of article type, abstracts, and keywords, 3994 articles were excluded because of irrelevant topics or because they were literature reviews, conference abstracts, editorial letters, or irrelevant studies. Full-text assessment was conducted on the remaining 951 articles. Subsequently, 802 articles were excluded as they did not meet the inclusion criteria. Finally, a quality assessment was performed on the remaining articles. We conducted a meta-analysis on the risk factors for PPM implantation within 30 days of TAVR, including only those factors that were evaluated in three or more studies. Fig. 1 shows the PRISMA flow diagram.

### 3.2 Study Characteristics

Ultimately, we included 82 studies (Table 1, Ref. [4, 7, 8, 9, 10, 11, 12, 13, 14, 15, 16, 17, 18, 19, 20, 21, 22, 23, 24, 25, 26, 27, 28, 29, 30, 31, 32, 33, 34, 35, 36, 37, 38, 39, 40, 41, 42, 43, 44, 45, 46, 47, 48, 49, 50, 51, 52, 53, 54, 55, 56, 57, 58, 59, 60, 61, 62, 63, 64, 65, 66, 67, 68, 69, 70, 71, 72, 73, 74, 75, 76, 77, 78, 79, 80, 81, 82, 83, 84, 85, 86]) involving 124,808 patients. Among these, 21,919 (17.5%) required PPM implantation within 30 days of TAVR. A total of 29,443 patients (23.6%) received self-expanding prostheses, while 42,414 (34.0%) received balloon-dilated prostheses. The remaining patients either did not have the specific valve type reported or were treated with other types of valves. The average PPM implantation rate was 20.4% for self-expanding prostheses and 12.8% for balloon-dilated prostheses. The average age of the included population was 82 ± 3 years, and 50.6% were males. The mean Society of Thoracic Surgeons risk score was 4.6 ± 4.5, and the average body mass index (BMI) was 26.5 ± 1.1 kg/m^2^. Additionally, 83.0% and 31.6% of the patients had hypertension and diabetes, respectively, and 52.4%, 23.3%, 28.3%, and 33.6% had a history of coronary artery disease, chronic obstructive pulmonary disease, atrial fibrillation, and chronic kidney disease, respectively.

**Table 1.** **Summary of included studies in the meta-analysis**.

First author	Publication year	Study type	Quality score	Total sample size (n)	Event sample size (n)	Mean age	Gender (male %)	STS Score	30-day all-cause mortality (%)
Kim, W. K. [17]	2018	Prospective Cohort	8/9	500	51	82.1	0.35	4.4 [3.1–6.6]	3.3
Abramowitz, Y. [18]	2016	Retrospective Cohort	7/9	582	69	82.0 ± 8.4	0.61	7.8 ± 4.7	2.7
Dhakal, B. P. [19]	2020	Retrospective Cohort	9/9	176	25	80 ± 8.5	0.6	5.7 ± 3.3	NA
Ahmad, M. [13]	2019	Retrospective Cohort	7/9	269	17	79.5 ± 8.7	0.51	6.2 ± 5.9	NA
Ko, E. [20]	2022	Prospective Cohort	7/9	676	58	79.8 ± 5.4	0.50	3.9 ± 2.9	2
Spargias, K. [21]	2013	Prospective Cohort	7/9	126	27	80 ± 8	0.41	7.0 ± 5.2	1
Corcione, N. [22]	2021	Prospective Cohort	7/9	3075	401	79.8 ± 5.4	0.50	NA	2.3
Stankowski, T. [23]	2021	Retrospective Cohort	9/9	148	9	80.5 ± 5.5	0.50	NA	2
Folliguet, T. A. [24]	2019	Prospective Cohort	7/9	11,033	1689	83.1 ± 7.0	0.48	NA	6.3
Marie, B. [25]	2021	Retrospective Cohort	7/9	500	60	81.0 ± 7.3	0.54	NA	1.2
Barki, M. [26]	2022	Retrospective Cohort	7/9	166	28	82.7 ± 6.1	0.57	3.7 ± 2.3	2.4
Kroon, H. G. [27]	2022	Prospective Cohort	9/9	368	74	80 [74–84]	0.53	4.3 [2.8–6.3]	4
Auffret, V. [28]	2017	Retrospective Cohort	9/9	3527	573	82 ± 8	0.50	6.9 ± 4.1	7.2
Okuno, T. [29]	2021	Prospective Cohort	7/9	875	186	81.9 ± 6.3	0.48	5.39 ± 3.62	3.4
Grubb, K. J. [7]	2023	Prospective Cohort	9/9	504	46	78.7 ± 6.6	0.54	3.0 ± 2.4	0.6
Nazif, T. M. [30]	2014	Prospective Cohort	8/9	1151	39	83.7 ± 7.3	0.43	11.3 ± 3.5	3.6
Haouzi, A. [31]	2022	Prospective Cohort	9/9	181	21	77.9 ± 9.1	0.62	3.49 ± 2.98	3.8
Tham, J. L. M. [32]	2020	Retrospective Cohort	7/9	151	27	83.6 ± 4.9	0.46	4.44 ± 3.8	2.6
Schofer, N. [33]	2018	Retrospective Cohort	7/9	273	62	80.6 ± 7.3	0.50	5.6 ± 5.2	2.6
Sawaya, F. J. [34]	2016	Retrospective Cohort	9/9	790	87	82.8 ± 7.1	0.52	5.3 ± 3.5	6.3
Abdel-Wahab, M. [35]	2014	RCT	Low Risk	241	64	80.8 ± 11.4	0.37	5.9 ± 3.4	4.6
Vlastra, W. [36]	2019	Prospective Cohort	8/9	12,831	1730	81 ± 7	0.42	6.4 ± 5.2	5.5
Thiele, H. [37]	2020	RCT	Low Risk	447	90	81.6 ± 5.5	0.49	4.8 [2.9–9.8]	2.7
Lak, H. M. [38]	2022	Retrospective Cohort	7/9	468	23	80.0 ± 8.2	0.59	5.3 ± 3.6	1.5
Abramowitz, Y. [39]	2017	Retrospective Cohort	9/9	761	34	82.1 ± 8.8	0.60	7.1 ± 5.4	3.3
Schewel, D. [40]	2018	Retrospective Cohort	8/9	563	61	81.2 ± 6.9	0.44	5.9 [3.4–8.0]	9.9
Simonato, M. [41]	2019	Retrospective Cohort	7/9	113	7	76.5 ± 9.7	0.66	8 ± 7.6	NA
Asmarats, L. [42]	2023	Prospective Cohort	7/9	85	19	81.7 ± 6.4	0.27	4.2 ± 2.8	2.4
Maeno, Y. [11]	2017	Retrospective Cohort	7/9	240	35	82.4 ± 7.3	0.51	5.2 ± 2.4	NA
Kiefer, N. J. [43]	2019	Retrospective Cohort	7/9	378	50	83.0 ± 8.6	0.49	7.1 ± 5.4	NA
Kroon, H. G. [44]	2022	Prospective Cohort	8/9	362	74	80 [73–84]	0.54	4.2 [2.8–6.3]	2.8
Mendiz, O. A. [45]	2021	Retrospective Cohort	7/9	257	28	79.7 ± 7.6	0.50	5.9 ± 2.5	3.5
Hokken, T. W. [9]	2022	Retrospective Cohort	9/9	1811	275	81.9 [77.2–85.4]	0.54	3.2 [2.1–5.0]	NA
Mesnier, J. [46]	2021	Retrospective Cohort	7/9	1177	323	80.8 ± 9.2	0.53	NA	5.7
Kim, K. [47]	2022	Retrospective Cohort	7/9	364	7	80.8 ± 5.4	0.46	5.9 ± 5.8	10.4
Ternacle, J. [48]	2021	Prospective Cohort	7/9	495	21	73.2 ± 5.8	0.64	NA	0.4
Fischer, Q. [49]	2018	Prospective Cohort	8/9	3404	529	81.0 ± 8.1	0.46	5.5 ± 3.2	5.7
Ojeda, S. [50]	2020	Retrospective Cohort	8/9	345	60	79 ± 6	0.46	NA	NA
Rajah, F. T. [51]	2022	Retrospective Cohort	8/9	170	48	76 [72–83]	0.57	NA	NA
Al-Azzam, F. [52]	2017	Retrospective Cohort	9/9	300	59	81.1 ± 8.4	0.55	7.6 [5.3–10.6]	NA
Hamdan, A. [8]	2015	Prospective Cohort	9/9	73	21	79.8 ± 6.9	0.45	NA	NA
Kooistra, N. H. M. [53]	2020	Prospective Cohort	9/9	2804	341	82 [77–85]	0.45	NA	NA
Useini, D. [54]	2022	Prospective Cohort	7/9	103	19	82.7 ± 4.6	0.44	3.7 ± 1.7	3.9
Chamandi, C. [55]	2019	Prospective Cohort	7/9	1020	157	80.6 ± 7.5	0.57	NA	2.9
Collas, V. M. [56]	2015	Prospective Cohort	7/9	861	113	83 [79–87]	0.47	NA	9
Rheude, T. [57]	2022	Retrospective Cohort	7/9	1612	110	82 [79–85]	0.48	NA	NA
Pellegrini, C. [58]	2019	Retrospective Cohort	7/9	709	115	81 ± 6	0.55	NA	NA
Guzel, T. [59]	2023	Retrospective Cohort	8/9	281	23	79.0 ± 6.3	0.45	8.55 ± 2.7	6.3
Habertheuer, A. [60]	2021	Prospective Cohort	7/9	563	78	82 [78–86]	0.57	NA	1.9
Dumonteil, N. [61]	2019	Prospective Cohort	7/9	1544	207	82	0.51	6.9	2
Hamdan, A. [62]	2021	Retrospective Cohort	9/9	134	18	77.0 ± 8.8	0.61	NA	NA
Pascual, I. [63]	2022	Prospective Cohort	8/9	444	54	82.4 ± 7.6	0.52	4.5 ± 2.4	1.8
Toutouzas, K. [64]	2019	RCT	Low Risk	171	41	81.7 ± 7.2	0.53	NA	0
Gama, F. [16]	2022	Prospective Cohort	9/9	273	57	84 [80–87]	0.39	NA	NA
Leclercq, F. [65]	2020	RCT	Low Risk	236	29	NA	NA	NA	1.7
Maier, O. [66]	2022	Retrospective Cohort	7/9	759	35	81.6 ± 5.6	0.49	4.9 ± 3.9	0.1
Chiam, P. T. L. [67]	2021	Retrospective Cohort	7/9	873	82	80 ± 7.2	0.46	NA	4.9
Havakuk, O. [68]	2016	Retrospective Cohort	7/9	324	81	83.2 ± 6.6	0.42	NA	2.5
Bernhard, B. [69]	2022	Retrospective Cohort	7/9	2213	453	82.1 ± 6.1	0.49	NA	3.3
Doldi, P. M. [70]	2022	Retrospective Cohort	7/9	122	25	83.2 ± 6.6	0.80	3.3 [2.2–4.6]	0.8
Siontis, G. C. M. [10]	2014	Retrospective Cohort	9/9	353	89	82.0 ± 7.4	0.54	14.4 ± 10.2	NA
Kim, W. J. [71]	2015	Retrospective Cohort	8/9	117	23	81.2 ± 5.1	0.51	NA	NA
Maan, A. [72]	2015	Retrospective Cohort	9/9	110	31	83.6 ± 7.1	0.54	4.04 [1.40–26.96]	NA
Monteiro, C. [73]	2017	Retrospective Cohort	8/9	670	135	NA	0.59	NA	25.8
Rampat, R. [74]	2017	Retrospective Cohort	7/9	201	64	81.2 ± 7.7	0.51	NA	NA
van Gils, L. [75]	2017	Retrospective Cohort	8/9	306	126	83 ± 7	0.63	6.3 [4.1–10.2]	7
Iacovelli, F. [76]	2018	Retrospective Cohort	9/9	86	8	81.7 ± 5.0	0.42	20.23 ± 14.62	0
Costa, G. [4]	2019	Prospective Cohort	9/9	1116	145	80.9 ± 5.3	0.42	4.4 ± 3.4	4.7
Jilaihawi, H. [77]	2019	Retrospective Cohort	9/9	248	24	83.2 ± 6.9	0.57	6.0 ± 2.9	1.2
Katchi, F. [15]	2019	Retrospective Cohort	9/9	136	51	84 ± 8	0.47	6 [4–8]	NA
Meduri, C.U. [78]	2019	RCT	Low Risk	912	245	82 ± 8	0.49	NA	8.4
Bisson, A. [14]	2020	Retrospective Cohort	9/9	49201	11010	82.4 ± 7	0.51	NA	3.5
Droppa, M. [79]	2020	Retrospective Cohort	9/9	1745	191	80.6 ± 6.9	0.49	NA	2
Du, F. [80]	2020	Retrospective Cohort	8/9	256	38	76.5 ± 6.1	0.42	7.1 ± 5.9	3.3
Krishnaswamy, A. [81]	2020	Retrospective Cohort	9/9	284	19	81.4	0.54	5.57 ± 3.83	0.4
Eliav, R. [82]	2021	Retrospective Cohort	8/9	338	83	NA	0.49	NA	3.8
Nai Fovino, L. [12]	2021	Retrospective Cohort	7/9	728	112	81.2 [77.9–84.7]	0.54	4.06 [2.56–7.45]	NA
Hokken, T.W. [83]	2021	Retrospective Cohort	8/9	653	120	80.6 [74.7–84.8]	0.52	3.0 [1.9–4.8]	NA
Nicolas, J. [84]	2021	Retrospective Cohort	8/9	922	120	82.4 ± 0.2	NA	NA	NA
Hioki, H. [85]	2022	Retrospective Cohort	9/9	754	31	85 [82–88]	0.29	6.60 [4.58–9.92]	NA
Pascual, I. [86]	2022	Prospective Cohort	9/9	226	40	83.5 ± 6.0	0.60	NA	5.3
Pascual, I. [63]	2022	Prospective Cohort	7/9	444	79	82.4 ± 7.6	0.52	4.5 ± 2.4	4.2

RCT, Randomized controlled trial; STS, Society of Thoracic Surgeons; NA, not available.

### 3.3 Baseline Patient Factors

**Fig. 2.**
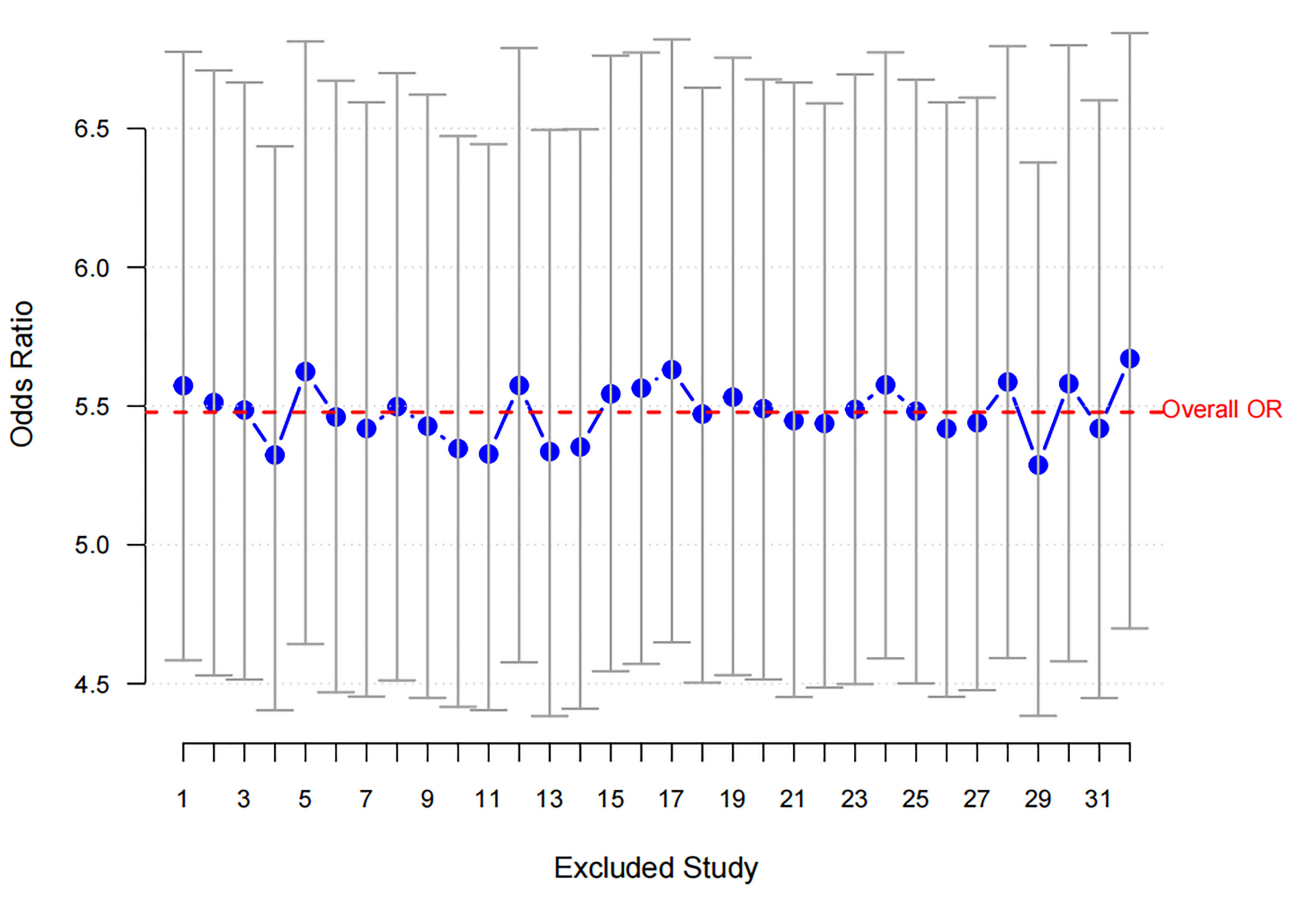
**Sensitivity analysis for RBBB**. The blue dots represent the ORs obtained after excluding each study, with the vertical lines indicating the 95% confidence intervals. The red dashed line represents the overall OR, showing consistent results across study exclusions. RBBB, Right Bundle Branch Block; OR, odds ratio.

**Fig. 3.**
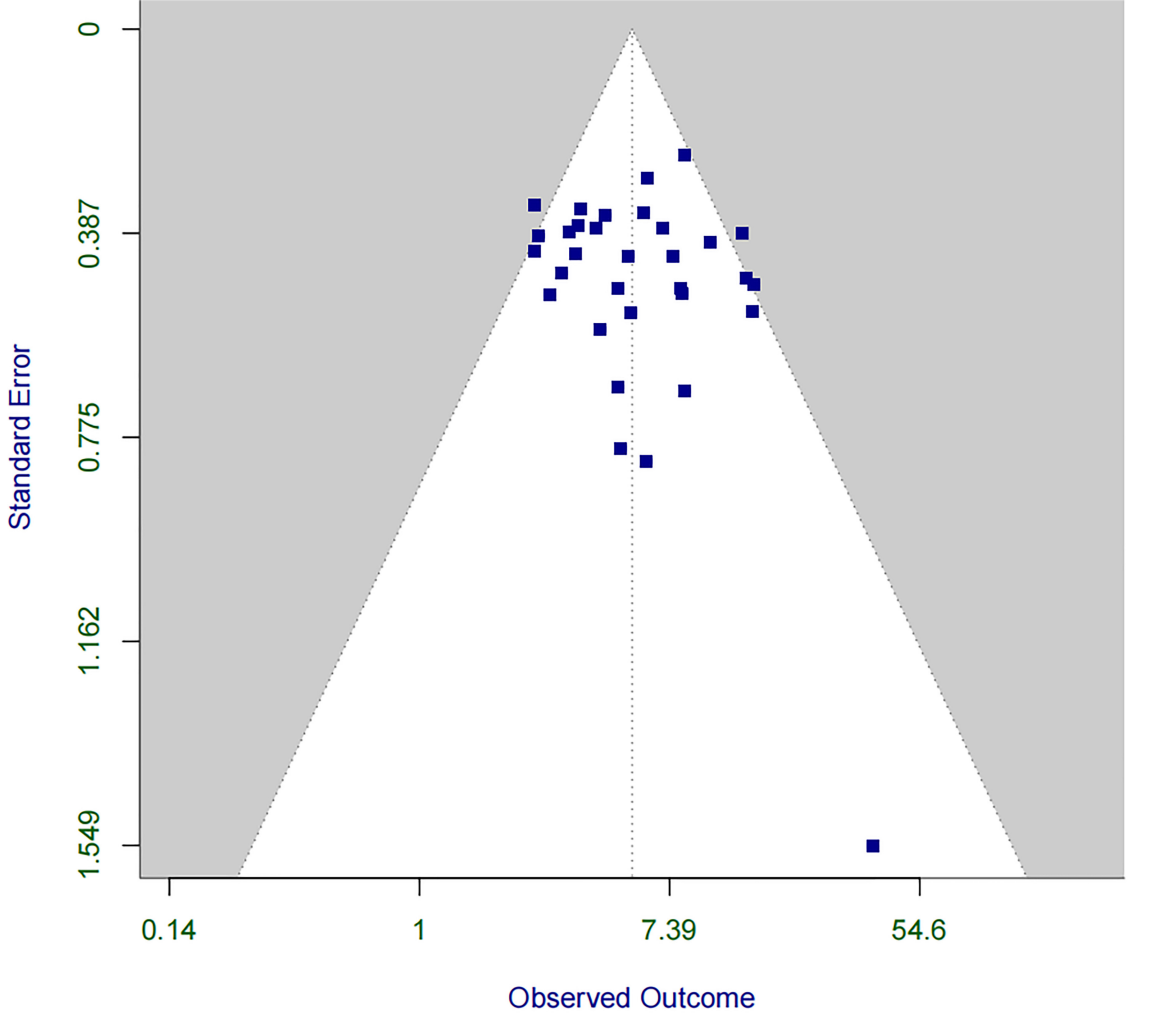
**Funnel plot for assessing publication bias in the association between RBBB and PPM implantation within 30 days post-TAVR**. PPM, permanent pacemaker; TAVR, transcatheter aortic valve replacement.

Several baseline patient factors were significantly associated with PPM implantation. RBBB showed a strong association with higher odds of PPM implantation (OR = 5.48, 95% CI: 4.52–6.64, *p* < 0.0001), with moderate heterogeneity (*I*^2^ = 37.74). Sensitivity analysis (Fig. 2) confirmed the robustness of this finding as the exclusion of individual studies did not substantially alter the overall OR, which remained between 5.0 and 6.0. Furthermore, we used funnel plots to assess publication bias and found that, although some asymmetry was observed, there was no significant evidence of publication bias for RBBB (Fig. 3). Additional funnel plots and sensitivity analyses for other risk factors are available in the **Supplementary Materials**. The first-degree AVB was also significantly associated with PPM implantation (OR = 2.30, 95% CI: 1.82–2.90, *p* < 0.0001), with no observed heterogeneity (*I*^2^ = 0). There was no significant publication bias, and the sensitivity analysis confirmed the robustness of this association. Baseline left bundle branch block (LBBB) showed no significant association (OR = 1.43, 95% CI: 0.67–3.07, *p* = 0.3599), but had high heterogeneity (*I*^2^ = 78.15). There was no significant publication bias; however, sensitivity analysis indicated poor robustness. Longer membranous septum (MS) length was significantly associated with PPM implantation (OR = 0.78, 95% CI: 0.66–0.93, *p* = 0.0065), with moderate heterogeneity (*I*^2^ = 78.13). There was no significant publication bias, and the sensitivity analysis confirmed the robustness of this association. Mitral annular calcification (MAC) was not significantly associated with PPM implantation (OR = 1.06, 95% CI: 0.75–1.49, *p* = 0.7560). Potential publication bias was observed, and the sensitivity analysis indicated poor robustness. Male gender was also not significantly associated with PPM implantation (OR = 0.88, 95% CI: 0.65–1.19, *p* = 0.3963). There was no significant publication bias; however, sensitivity analysis indicated poor robustness.

### 3.4 Procedural Factors

Several procedural factors have been identified as significant risk factors for PPM implantation. Increased implant depth per mm was significantly associated with higher odds of PPM implantation (OR = 1.20, 95% CI: 1.13–1.28, *p* < 0.0001) as well as low implant depth (OR = 1.18, 95% CI: 1.12–1.24, *p* < 0.0001), both showing low to no heterogeneity. There was no significant publication bias, and the sensitivity analysis confirmed the robustness of these associations. The use of self-expanding valve was significantly associated with increased odds of PPM implantation (OR = 2.59, 95% CI: 2.06–3.27, *p* < 0.0001), with substantial heterogeneity (*I*^2^ = 60.13). Potential publication bias was observed, but sensitivity analysis confirmed the robustness of this association.

Additionally, the use of the COT significantly reduced the risk of PPM implantation (OR = 0.45, 95% CI: 0.35–0.58, *p* < 0.0001), with no observed heterogeneity (*I*^2^ = 0). There was no significant publication bias, and the sensitivity analysis confirmed the robustness of this finding. The difference between MS length and implantation depth (ΔMSID) was also significantly associated with PPM implantation (OR, 1.36; 95% CI, 1.22–1.50, *p* < 0.0001), with high heterogeneity (*I*^2^ = 41.82). There was no significant publication bias, and the sensitivity analysis confirmed the robustness of this association. Predilation was another significant risk factor (OR = 1.37, 95% CI: 1.10–1.71, *p* = 0.0045), with no observed heterogeneity. Potential publication bias was noted, and the sensitivity analysis indicated poor robustness.

### 3.5 Post-Procedural Factors

Only one post-procedural risk factor met the inclusion criteria. New-onset LBBB was identified as a significant post-procedural risk factor for PPM implantation (OR = 2.26, 95% CI: 1.66–3.07, *p* < 0.0001), with no observed heterogeneity (*I*^2^ = 0). This finding highlights the importance of monitoring for new-onset LBBB after TAVR. However, it should be noted that a potential publication bias was observed, and the sensitivity analysis indicated a limited robustness of this association.

### 3.6 Temporal Trend Analysis

**Fig. 4.**
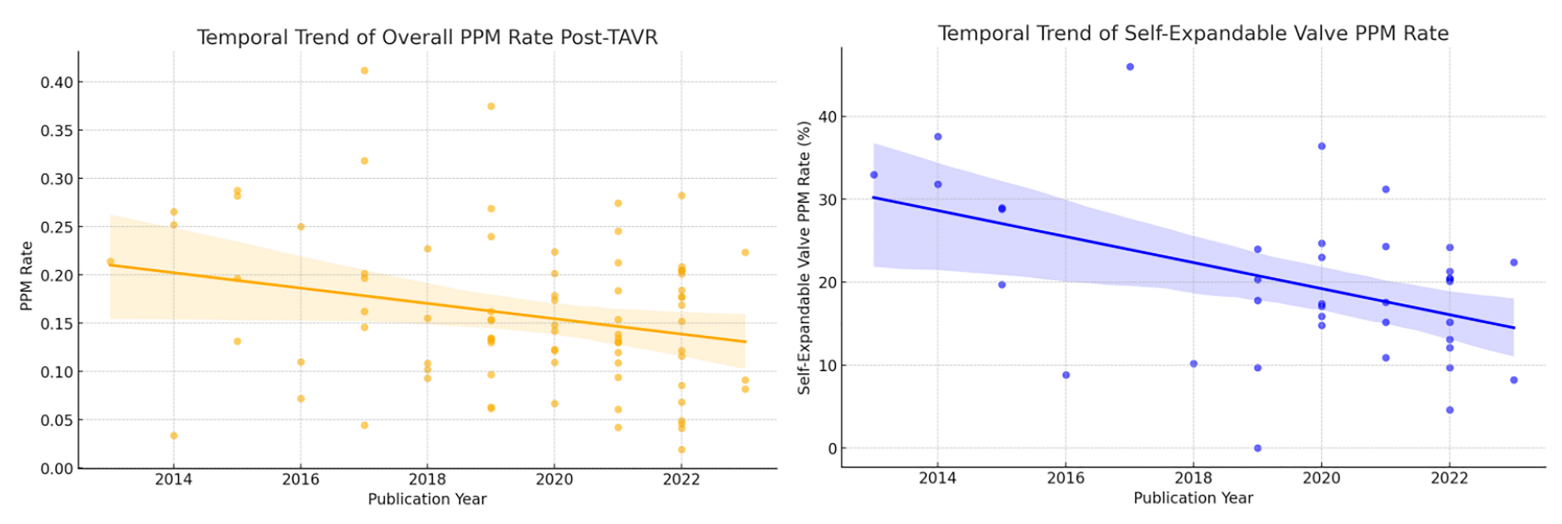
**Temporal trend in 30-Day PPM implantation rates after TAVR**.

A temporal trend analysis based on the year of publication was performed to evaluate changes in the 30-day PPM implantation rate following TAVR (Fig. 4). Meta-regression revealed a significant decreasing trend in overall PPM implantation rates over time (coefficient = –0.008, 95% CI: –0.015 to –0.001, *p* = 0.023). Specifically, a notable decline was observed in the self-expanding valve subgroup (coefficient = –1.571, 95% CI: –2.620 to –0.521, *p* = 0.004), whereas no significant temporal change was observed for the balloon-expandable valve subgroup (coefficient = –0.248, 95% CI: –1.408 to 0.911, *p* = 0.666).

### 3.7 Subgroup Analysis by Study Type

Subgroup analysis by study design was performed to investigate the potential impact of different study methodologies (RCT, prospective cohort, retrospective cohort) on reported risk factors for PPM implantation. The overall PPM rates did not significantly differ among these study types (Kruskal-Wallis test: statistic = 4.00, *p* = 0.261). Furthermore, subgroup analyses for specific risk factors (e.g., baseline RBBB, self-expanding valve use, increased implantation depth, baseline LBBB, low implant depth, MAC, and pre-dilation) did not reveal significant heterogeneity between RCTs and observational studies, with all risk factors demonstrating non-significant differences across different study designs (all *p* > 0.05).

## 4. Discussion

### 4.1 Overview

**Fig. 5.**
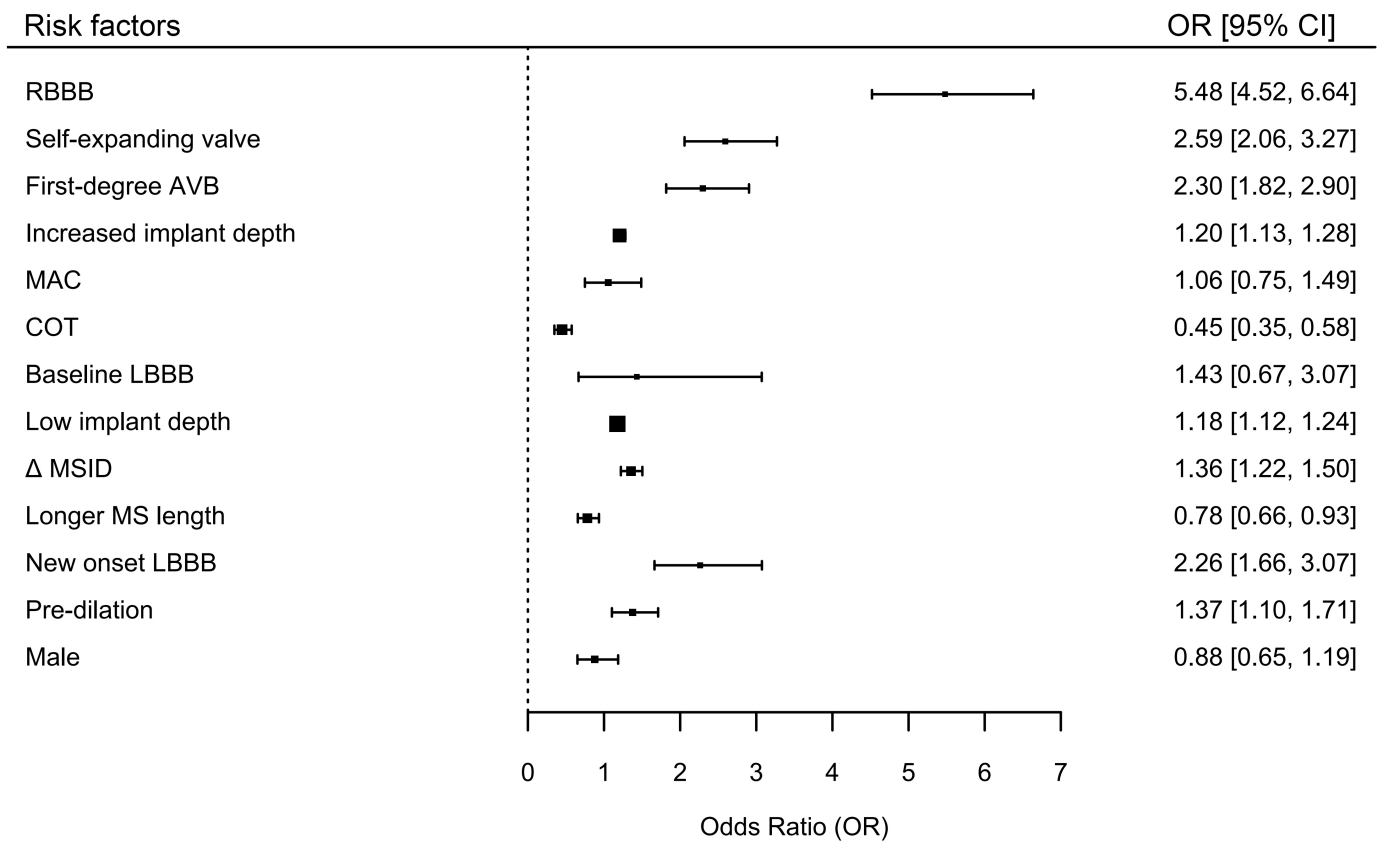
**Overall forest plot for various risk factors associated with PPM implantation within 30 days post-TAVR using random effects model**. SEV, Self-Expanding Valve; AVB, Atrioventricular Block; MAC, Mitral Annular Calcification; COT, Cusp Overlap Technique; LBBB, Left Bundle Branch Block; Δ MSID, Difference Between Membranous Septum Length and Implantation Depth; MS, Membranous Septum; New onset LBBB, New Onset Left Bundle Branch Block.

This study aimed to investigate the risk factors for PPM implantation within 30 days of TAVR to provide evidence for perioperative management [87]. Our findings indicated that baseline factors such as RBBB and first-degree AVB were associated with an increased risk of PPM implantation, whereas a longer MS length was associated with a lower risk. Procedural factors, including increased implant depth, low implant depth, use of self-expanding valves, and predilation, were associated with a higher risk of PPM implantation. Conversely, a lower ΔMSID and the use of the COT could reduce the risk of PPM implantation. Postoperative new-onset LBBB was a risk factor for increased PPM implantation. Sex, MAC, and baseline LBBB were not associated with PPM implantation. Fig. 5 illustrates the overall forest plot of various risk factors associated with PPM implantation within 30 days post-TAVR.

### 4.2 Baseline Risk Factors

Identifying high-risk patients for PPM implantation before intervention is crucial, as it can significantly influence the treatment strategy and patient’s prognosis. Our meta-analysis found that baseline RBBB increased the risk of PPM implantation by approximately 5.5 times, which is much higher than that of other risk factors. This increased risk was likely because valve implantation could easily damage the left bundle branch. If patients with a preexisting RBBB developed LBBB after TAVR, the incidence of chronic arrhythmias and high-grade AVB increased [6, 7]. However, our study found that the baseline LBBB was not associated with an increased need for PPM implantation after TAVR. This finding may further indicated why patients with a baseline RBBB were more prone to PPM implantation due to the damage to the LBBB caused by the valve deployment and positioning during the procedure. Additionally, first-degree AVB significantly increased the risk of PPM implantation, indicating that preoperative attention to conduction abnormalities was necessary.

Patients with longer MS had a lower risk of PPM implantation, which might be related to anatomical factors. Typically, the His bundle originates from the atrioventricular node, traverses the central fibrous body, and extends into the membranous septum, coursing below the junction of the noncoronary and right coronary cusps, with a total length of approximately 20 mm [88]. As the His bundle and left bundle branch are close to the aortic annulus, some conduction abnormalities during surgery are secondary to mechanical damage to the aortic root, leading to tissue inflammation, edema, or ischemia [89]. A longer MS suggests a greater distance from the annulus to the His bundle, reducing the likelihood of valve-induced compression and thus lowering the risk of conduction block [8, 9].

Interestingly, other meta-analyses have reported that male patients have a higher risk of PPM implantation after TAVR [10, 90], whereas some have found that female patients are more likely to develop new-onset LBBB [6]. However, in our study, which analyzed studies that adjusted for sex in multivariate analyses, we found that sex was not associated with the risk for PPM implantation. These results were consistent across the included studies, indicating no heterogeneity. Some studies have suggested that the higher risk in males may be due to the more frequent use of oversized valves and the higher prevalence of baseline comorbidities [91]. The effect of sex on the risk of PPM implantation remains controversial and warrants further investigation.

### 4.3 Procedural Risk Factors

Except for baseline factors, procedure-related risk factors were crucial for outcomes. Our findings were consistent with those of previous studies indicating that the use of self-expanding valves significantly increased the risk of PPM implantation after TAVR [6, 89, 90]. This increase might be due to mechanical damage or pressure exerted on the conduction system, leading to tissue inflammation, ischemia, edema, and subsequent conduction abnormalities [2, 11, 12, 92]. Similarly, predilation procedures also elevated the risk of conduction abnormalities by 1.37 times, although the funnel plot indicated potential publication bias for predilation. Predilation should not be performed routinely unless necessary. Valve implantation depth was another critical factor that influenced outcomes. As previously mentioned, the cardiac conduction system was associated with the MS length. Our meta-analysis indicated that valve implantation depth was closely related to the occurrence of conduction abnormalities, which supported the mechanism underlying these post-TAVR complications. Consequently, the measurement of ΔMSID has been proposed to predict the risk of PPM implantation. Both MS length and valve implantation depth could be measured, and our meta-analysis confirmed that ΔMSID was significantly associated with PPM implantation, which provided valuable insights into treatment strategies. Preoperative CT assessment of MS length allows calculation of ΔMSID, which guides individualized valve depth positioning.

In recent years, the COT has been proposed as a novel projection method for valve deployment. This technique involved overlapping the right and left coronary cusps to eliminate parallax, thereby facilitating accurate assessment of implantation depth and reducing the risk of conduction abnormalities [93]. Our study demonstrated that the COT significantly reduced the risk of PPM implantation after TAVR, with no heterogeneity observed among the included studies. COT is beneficial for high-risk patients, especially those with RBBB or first-degree AVB, to mitigate conduction injury.

### 4.4 Postoperative Risk Factors

Among the postoperative factors, only new-onset LBBB met the inclusion criteria. New-onset LBBB was common after TAVR, and we found that it increased the risk of PPM implantation by 2.26 times. Although one-third of patients could reach a resolution of LBBB within 30 days postoperatively as myocardial injury, inflammation, or edema subsided, it is important to note that new-onset LBBB implied the cardiac conduction system had been affected [94]. Therefore, monitoring for conduction block complications in these patients should be warranted, and continuous electrocardiogram monitoring might be reasonable for this patient group [87, 95]. However, it should be paid attention that potential publication bias was observed in our study and the robustness of the findings was limited, necessitating cautious interpretation and further research on the relationship between new-onset LBBB and the need for PPM implantation after TAVR.

### 4.5 Temporal Trend Analysis

The temporal trend analysis demonstrates a significant overall decline in the incidence of PPM implantation following TAVR over recent years, particularly pronounced in self-expanding valve cohorts. This trend likely reflects technological advancements, such as the adoption of the cusp overlap technique, improved procedural optimization, and iterative upgrades in valve design, all contributing to more precise valve positioning and reduced conduction disturbances [93]. However, no significant temporal change was identified in balloon-expandable valve cohorts, possibly indicating a plateau in technological advancements or a consistent patient selection approach in this subgroup.

### 4.6 Limitations

Our study has several limitations. First, as this is the first meta-analysis to focus on the perioperative period of TAVR, we excluded high-quality studies whose endpoints did not align with our criteria. Second, we only included studies that performed multivariate adjustments for risk factors to eliminate confounding effects. Consequently, compared with previous meta-analyses, only 13 factors met our criteria; however, this provided stronger evidence regarding the impact of these factors on PPM implantation after TAVR. Third, although some variables such as BMI [13, 14], choice of oversized valves [4], and changes in QRS duration [15, 16], have been identified in previous studies as potentially related to conduction abnormalities, we required a minimum of three studies for each variable to ensure reasonable evaluation. Owing to differences in definitions among studies, some variables were ultimately not included in our analysis [96]. Lastly, although the number of eligible RCTs included in our meta-analysis was limited, we employed rigorous quality assessment tools for observational studies and conducted subgroup analyses based on study design. The results showed that the estimated effects of risk factors were largely consistent across study types, thereby strengthening the reliability of our overall findings.

## 5. Conclusions

This study fills a critical evidence gap by systematically evaluating perioperative predictors of PPM implantation within 30 days post-TAVR—a pivotal timeframe for early clinical decision-making. Compared to prior meta-analyses that focused on long-term outcomes or mixed timeframes, our study provides actionable insights for immediate post-TAVR management. We identified several key risk factors for PPM implantation, including baseline RBBB, first-degree AVB, shorter MS, use of self-expanding valves, predilation, lower valve implantation positions, and new-onset LBBB. However, the use of COT and a lower ΔMSID appeared to have protective effects, findings that had not been previously emphasized in pooled analyses. These results underscore the importance of preoperative assessment and procedural planning to mitigate conduction disturbances and optimize patient outcomes.

## Data Availability

Data extracted from included studies, data used for all analyses, analytic code, and other materials used in the systematic review are available upon request from the corresponding author.

## Abbreviations

TAVR, Transcatheter aortic valve replacement; PPM, Permanent pacemaker; RBBB, Right bundle branch block; first-degree AVB, first-degree atrioventricular block; LBBB, Left bundle branch block; MS, Membranous septum; COT, Cusp overlap technique; ΔMSID, Difference between membranous septum length and implantation depth; OR, Odds ratio; CI, Confidence interval; COP, Cusp-overlap projection.

## Supplementary Material

Supplementary material associated with this article can be found, in the online version, at https://doi.org/10.31083/RCM39299.
