# Supplementary material for: Perioperative Risk Factors for Permanent Pacemaker Implantation After Transcatheter Aortic Valve Replacement: A Systematic Review and Meta-Analysis
Source: Rev Cardiovasc Med. 2025 Oct 23;26(10):39299. doi: 10.31083/RCM39299 (PMC12593737; doi:10.31083/RCM39299)
Supplement: Supplementary file 1 [file 2153-8174-26-10-39299-s1.zip › Supplementary Materials.pdf]

supplementary materials

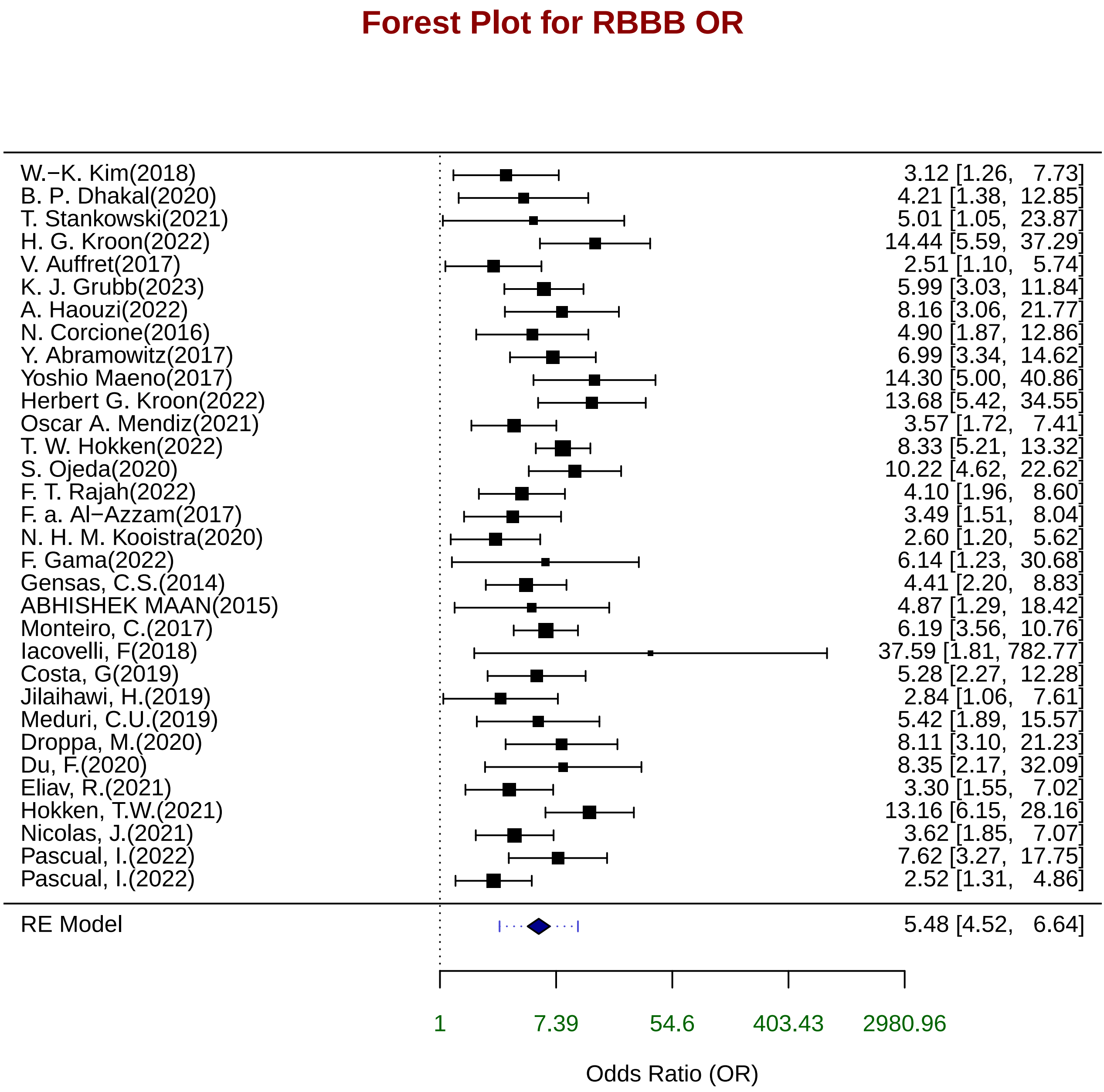
Forest Plot for RBBB OR
W−K. Kim(2018) 3.12 [1.26, 7.73]
B. P. Dhakal(2020) 4.21 [1.38, 12.85]
T. Stankowski(2021) 5.01 [1.05, 23.87]
H. G. Kroon(2022) 14.44 [5.59, 37.29]
V. Auffret(2017) 2.51 [1.10, 5.74]
K. J. Grubb(2023) 5.99 [3.03, 11.84]
A. Haouzi(2022) 8.16 [3.06, 21.77]
N. Corcione(2016) 4.90 [1.87, 12.86]
Y. Abramowitz(2017) 6.99 [3.34, 14.62]
Yoshio Maeno(2017) 14.30 [5.00, 40.86]
Herbert G. Kroon(2022) 13.68 [5.42, 34.55]
Oscar A. Mendiz(2021) 3.57 [1.72, 7.41]
T. W. Hokken(2022) 8.33 [5.21, 13.32]
S. Ojeda(2020) 10.22 [4.62, 22.62]
F. T. Rajah(2022) 4.10 [1.96, 8.60]
F. a. Al−Azzam(2017) 3.49 [1.51, 8.04]
N. H. M. Kooistra(2020) 2.60 [1.20, 5.62]
F. Gama(2022) 6.14 [1.23, 30.68]
Gensas, C.S.(2014) 4.41 [2.20, 8.83]
ABHISHEK MAAN(2015) 4.87 [1.29, 18.42]
Monteiro, C.(2017) 6.19 [3.56, 10.76]
Iacovelli, F(2018) 37.59 [1.81, 782.77]
Costa, G(2019) 5.28 [2.27, 12.28]
Jilaihawi, H.(2019) 2.84 [1.06, 7.61]
Meduri, C.U.(2019) 5.42 [1.89, 15.57]
Droppa, M.(2020) 8.11 [3.10, 21.23]
Du, F.(2020) 8.35 [2.17, 32.09]
Eliav, R.(2021) 3.30 [1.55, 7.02]
Hokken, T.W.(2021) 13.16 [6.15, 28.16]
Nicolas, J.(2021) 3.62 [1.85, 7.07]
Pascual, I.(2022) 7.62 [3.27, 17.75]
Pascual, I.(2022) 2.52 [1.31, 4.86]
RE Model 5.48 [4.52, 6.64]
1
7.39
54.6
403.43
2980.96
Odds Ratio (OR)

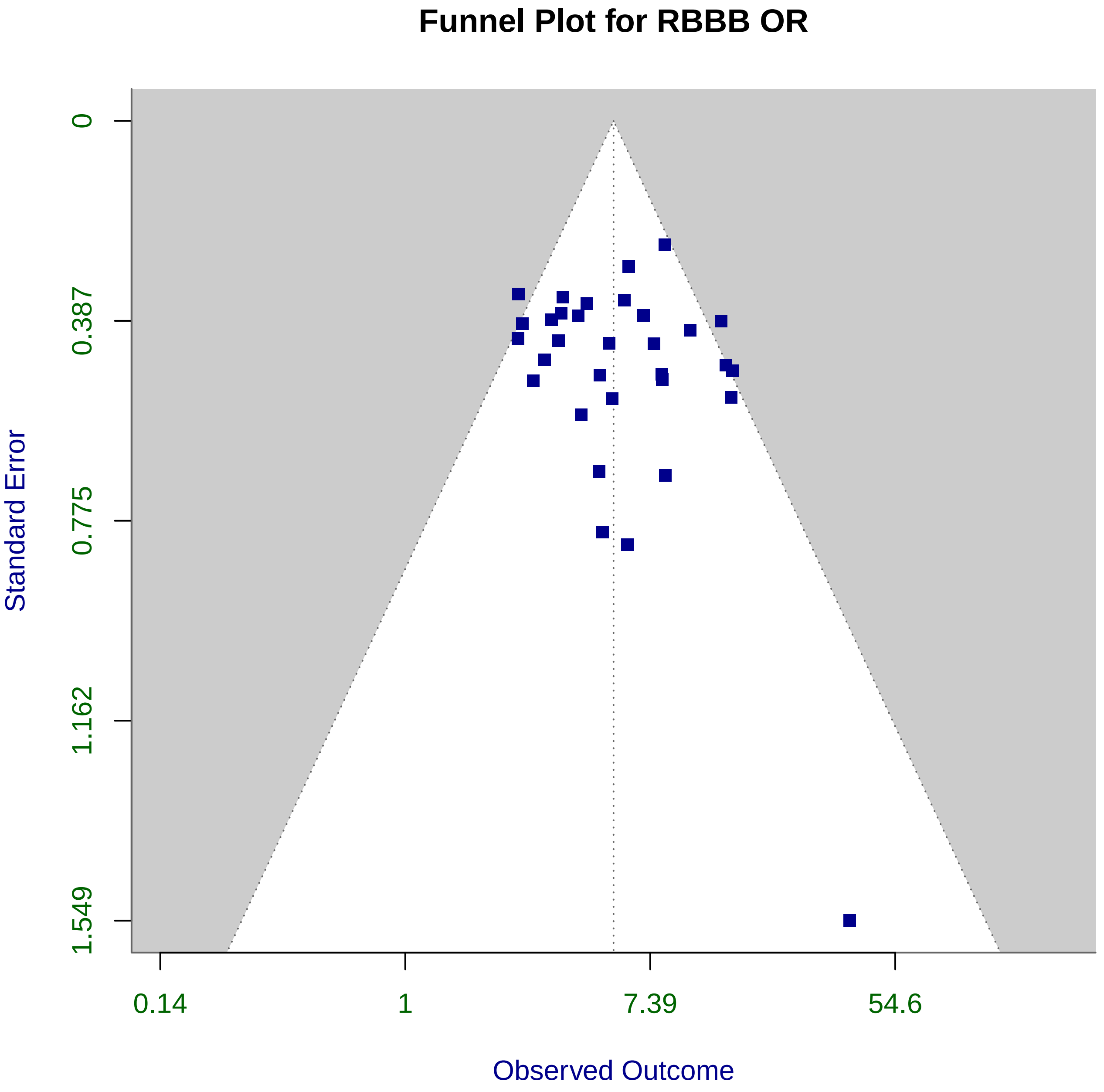
Funnel Plot for RBBB OR
Standard Error
0
0.387
0.775
1.162
1.549
0.14
1
7.39
54.6
Observed Outcome

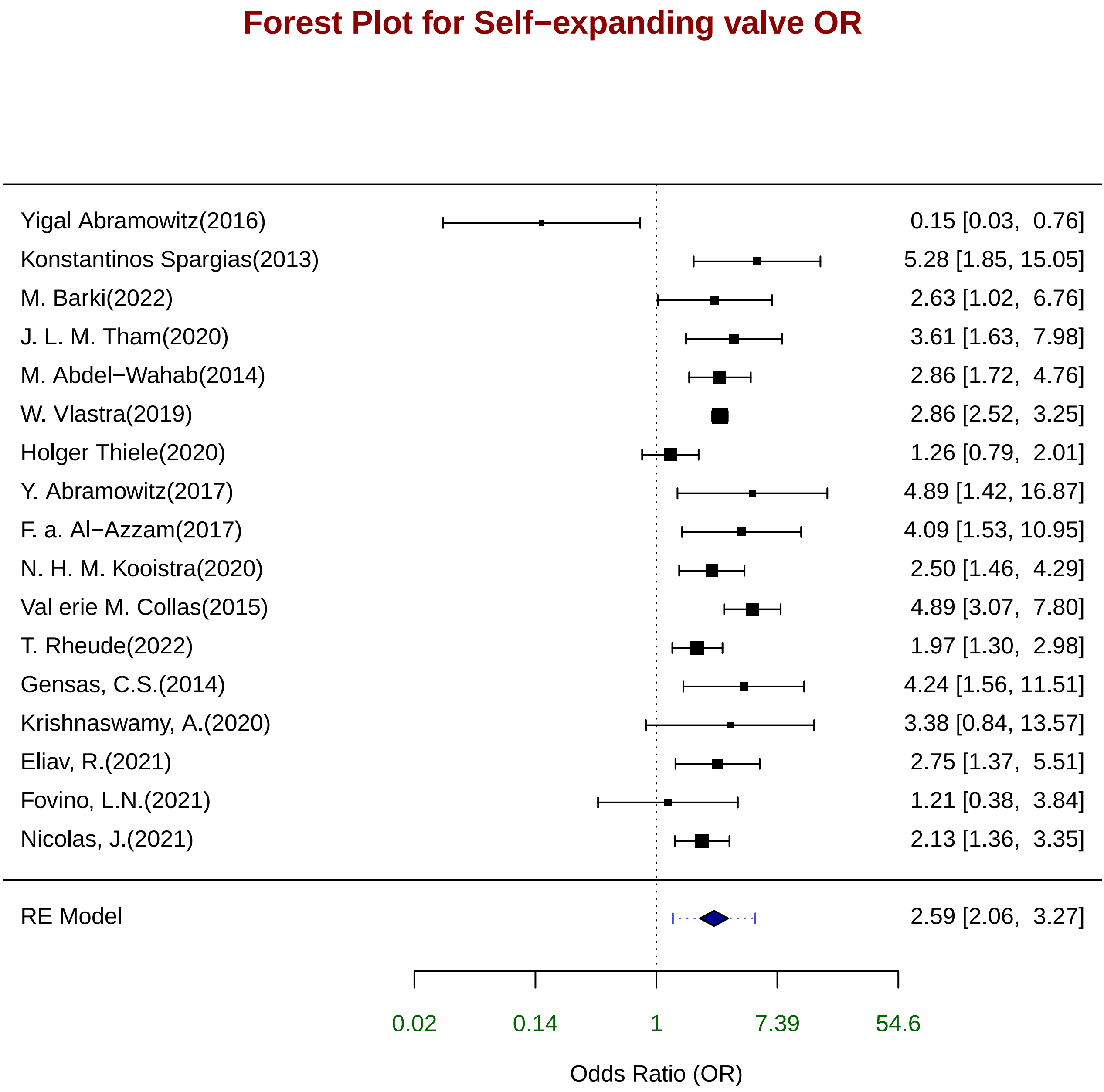
Forest Plot for Self−expanding valve OR
Yigal Abramowitz(2016) 0.15 [0.03, 0.76]
Konstantinos Spargias(2013) 5.28 [1.85, 15.05]
M. Barki(2022) 2.63 [1.02, 6.76]
J. L. M. Tham(2020) 3.61 [1.63, 7.98]
M. Abdel−Wahab(2014) 2.86 [1.72, 4.76]
W. Vlastra(2019) 2.86 [2.52, 3.25]
Holger Thiele(2020) 1.26 [0.79, 2.01]
Y. Abramowitz(2017) 4.89 [1.42, 16.87]
F. a. Al−Azzam(2017) 4.09 [1.53, 10.95]
N. H. M. Kooistra(2020) 2.50 [1.46, 4.29]
Val erie M. Collas(2015) 4.89 [3.07, 7.80]
T. Rheude(2022) 1.97 [1.30, 2.98]
Gensas, C.S.(2014) 4.24 [1.56, 11.51]
Krishnaswamy, A.(2020) 3.38 [0.84, 13.57]
Eliav, R.(2021) 2.75 [1.37, 5.51]
Fovino, L.N.(2021) 1.21 [0.38, 3.84]
Nicolas, J.(2021) 2.13 [1.36, 3.35]
RE Model 2.59 [2.06, 3.27]
0.02
0.14
1
7.39
54.6
Odds Ratio (OR)

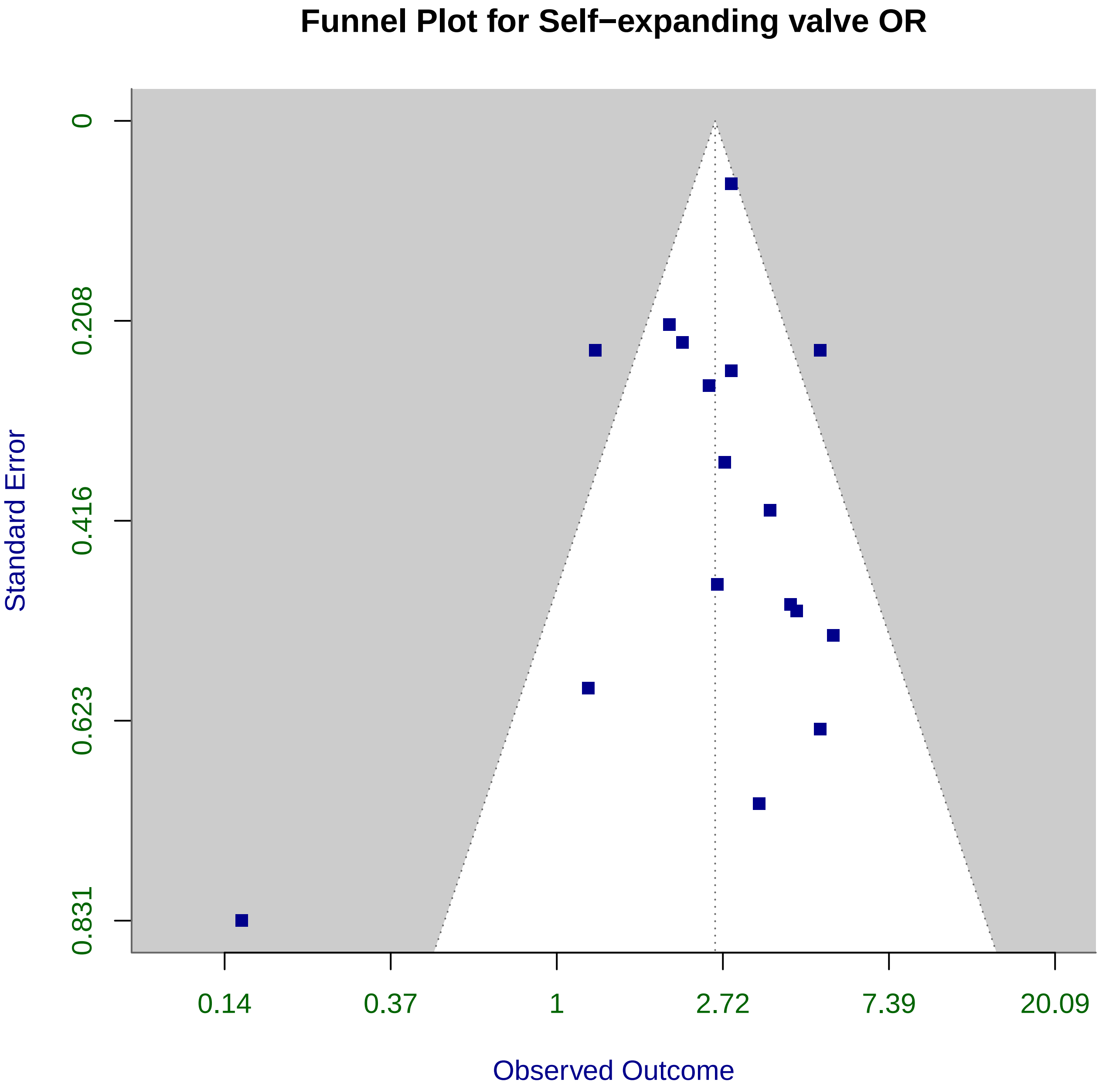
Funnel Plot for Self−expanding valve OR
Standard Error
0
0.208
0.416
0.623
0.831
0.14
0.37
1
2.72
7.39
20.09
Observed Outcome

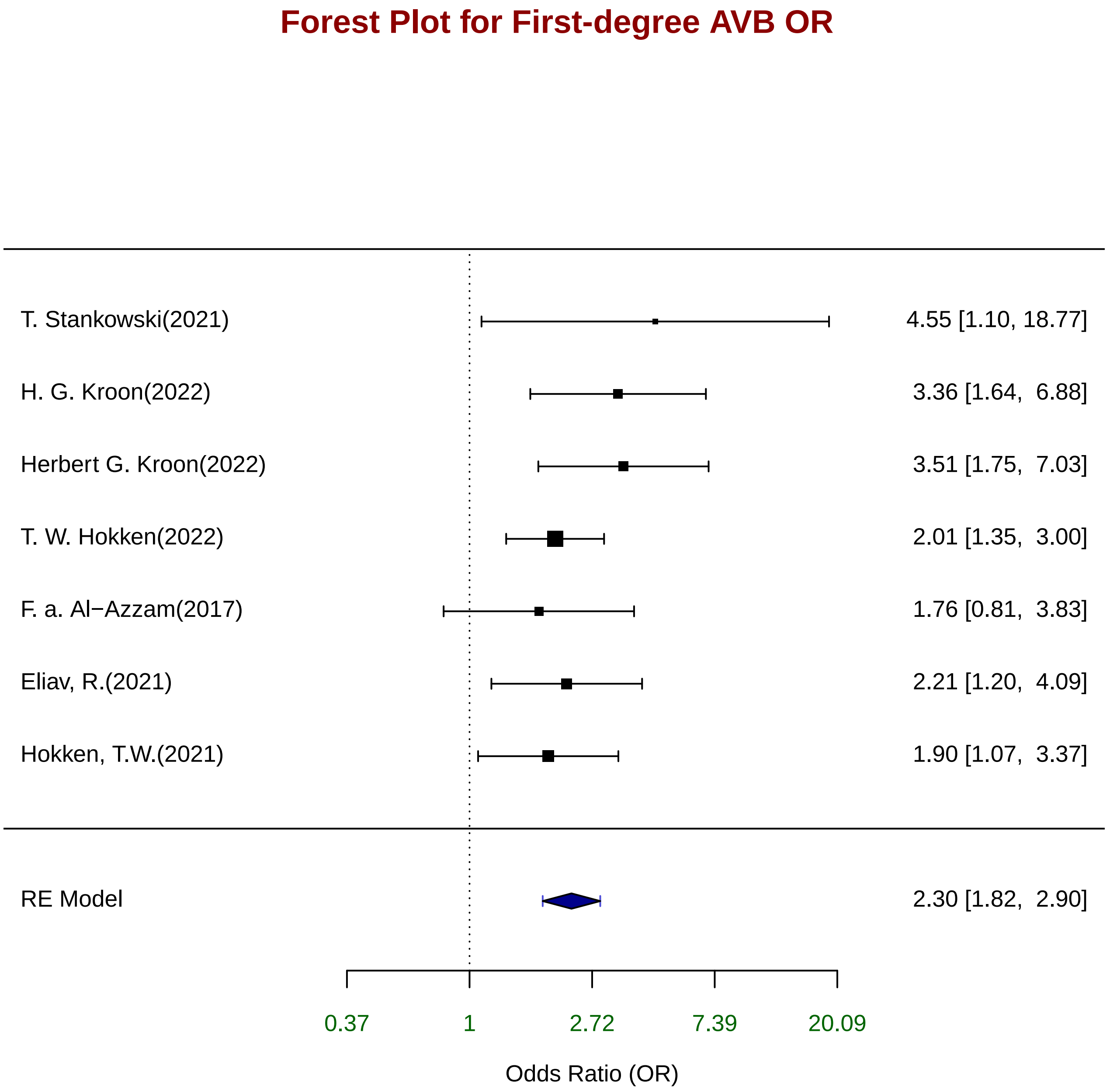
Forest Plot for First-degree AVB OR
T. Stankowski(2021)
4.55 [1.10, 18.77]
H. G. Kroon(2022)
3.36 [1.64, 6.88]
Herbert G. Kroon(2022)
3.51 [1.75, 7.03]
T. W. Hokken(2022)
2.01 [1.35, 3.00]
F. a. Al−Azzam(2017)
1.76 [0.81, 3.83]
Eliav, R.(2021)
2.21 [1.20, 4.09]
Hokken, T.W.(2021)
1.90 [1.07, 3.37]
RE Model
2.30 [1.82, 2.90]
0.37
1
2.72
7.39
20.09
Odds Ratio (OR)

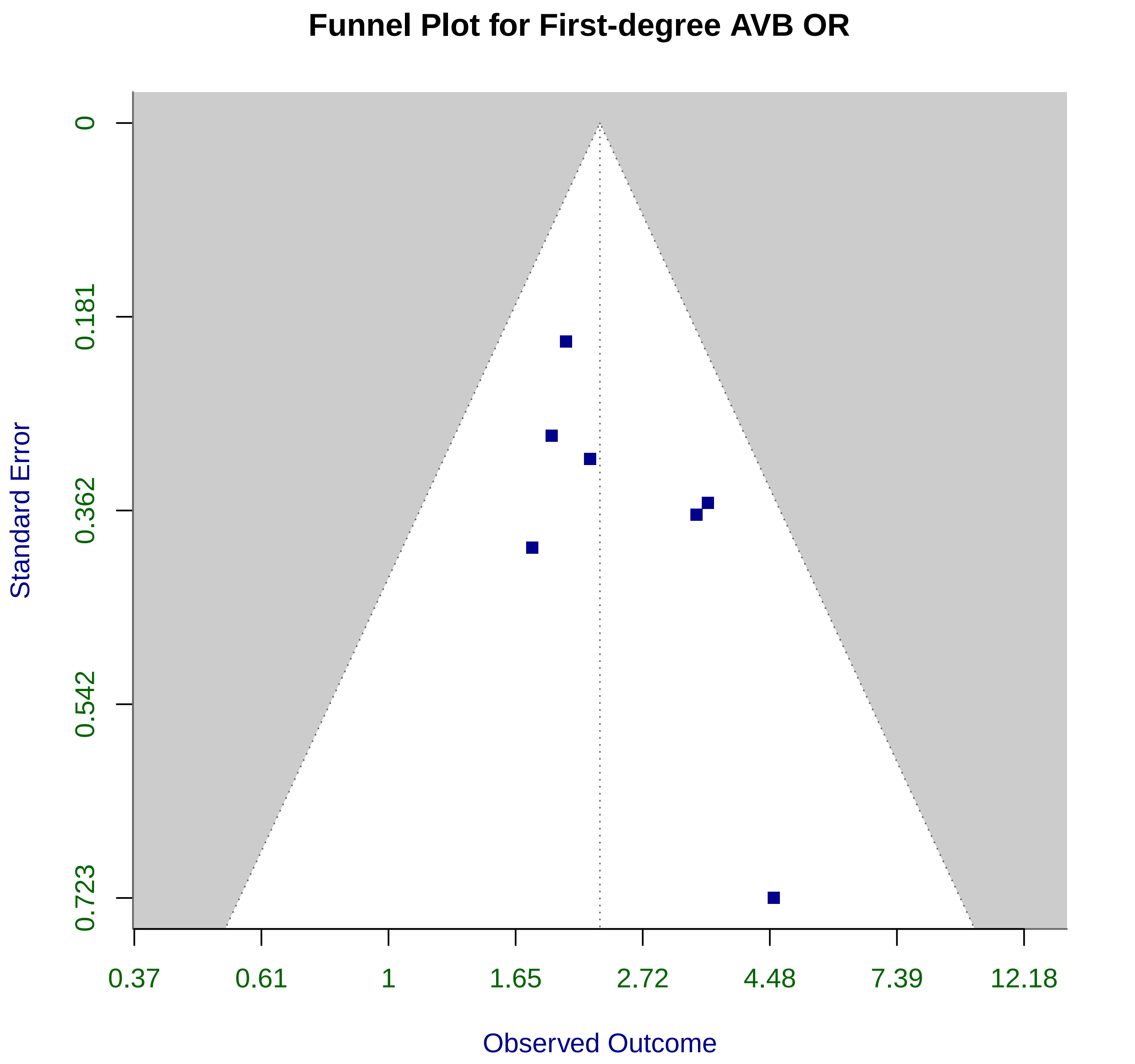

Funnel Plot for First-degree AVB OR
Standard Error
0
0.181
0.362
0.542
0.723
0.37
0.61
1
1.65
2.72
4.48
7.39
12.18
Observed Outcome

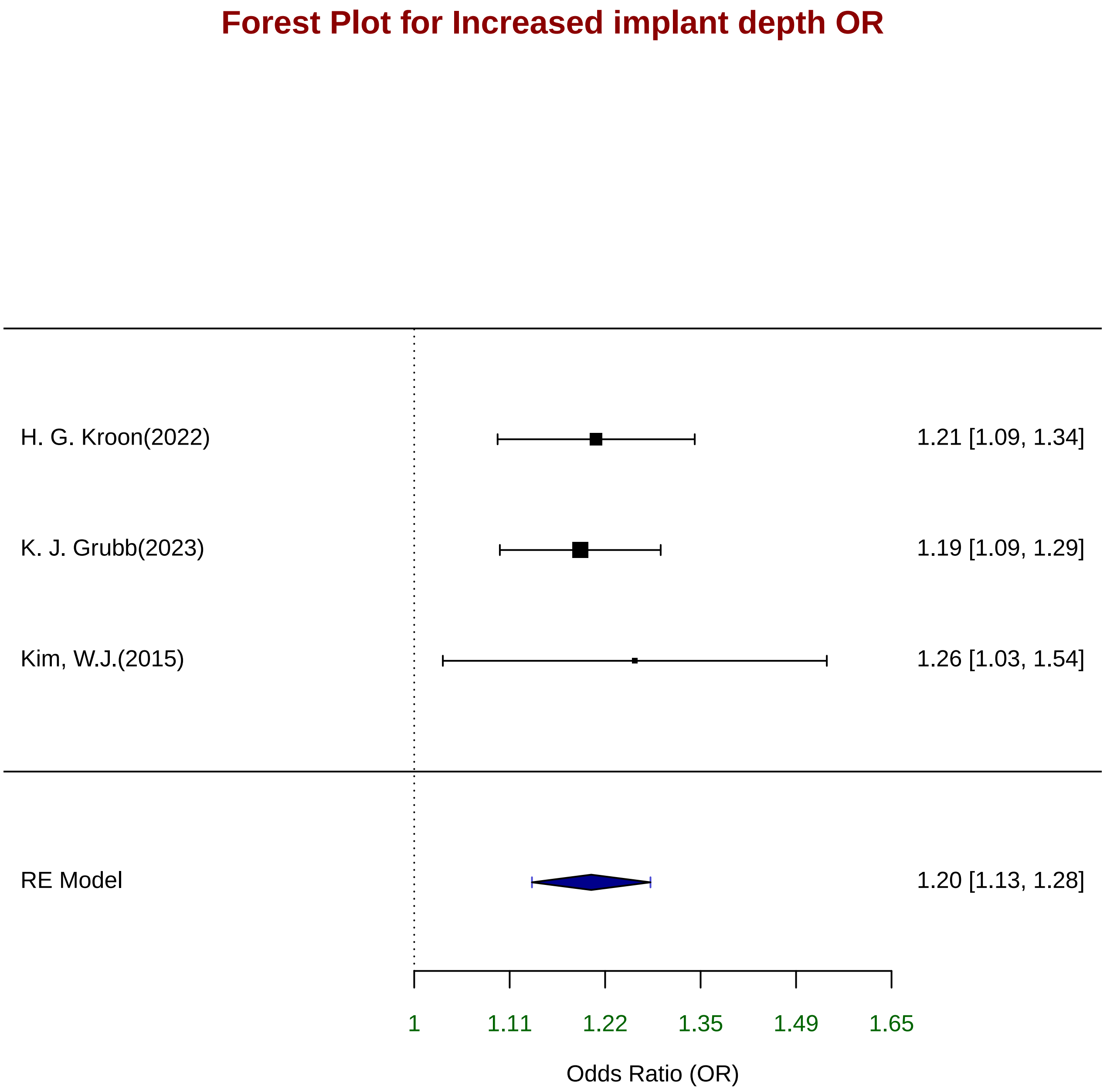
Forest Plot for Increased implant depth OR
H. G. Kroon(2022)
1.21 [1.09, 1.34]
K. J. Grubb(2023)
1.19 [1.09, 1.29]
Kim, W.J.(2015)
1.26 [1.03, 1.54]
RE Model
1.20 [1.13, 1.28]
1
1.11
1.22
1.35
1.49
1.65
Odds Ratio (OR)

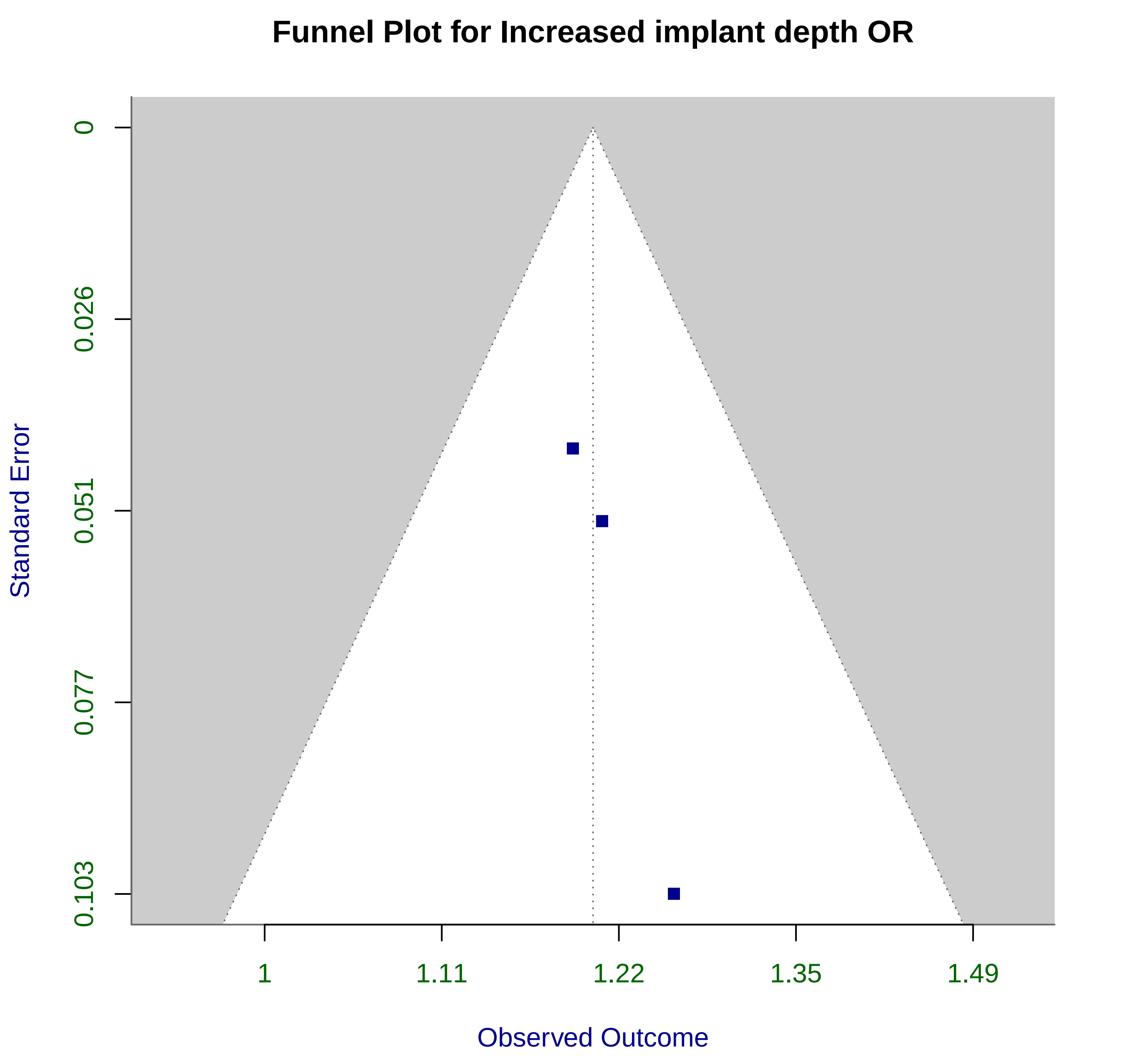
Funnel Plot for Increased implant depth OR
Standard Error
0
0.026
0.051
0.077
0.103
1
1.11
1.22
1.35
1.49
Observed Outcome

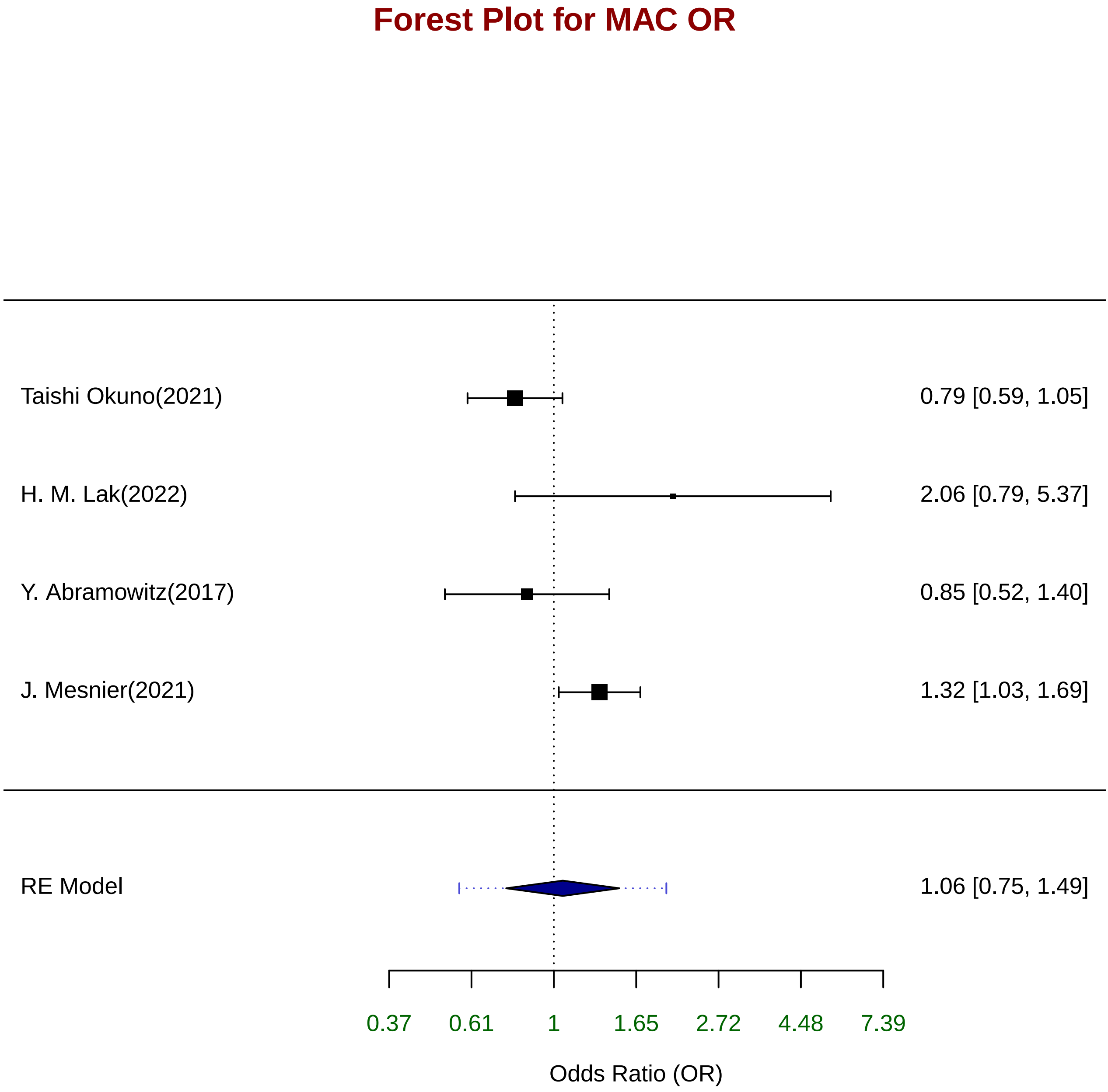

Forest Plot for MAC OR
Taishi Okuno(2021)
0.79 [0.59, 1.05]
H. M. Lak(2022)
2.06 [0.79, 5.37]
Y. Abramowitz(2017)
0.85 [0.52, 1.40]
J. Mesnier(2021)
1.32 [1.03, 1.69]
RE Model
1.06 [0.75, 1.49]
0.37
0.61
1
1.65
2.72
4.48
7.39
Odds Ratio (OR)

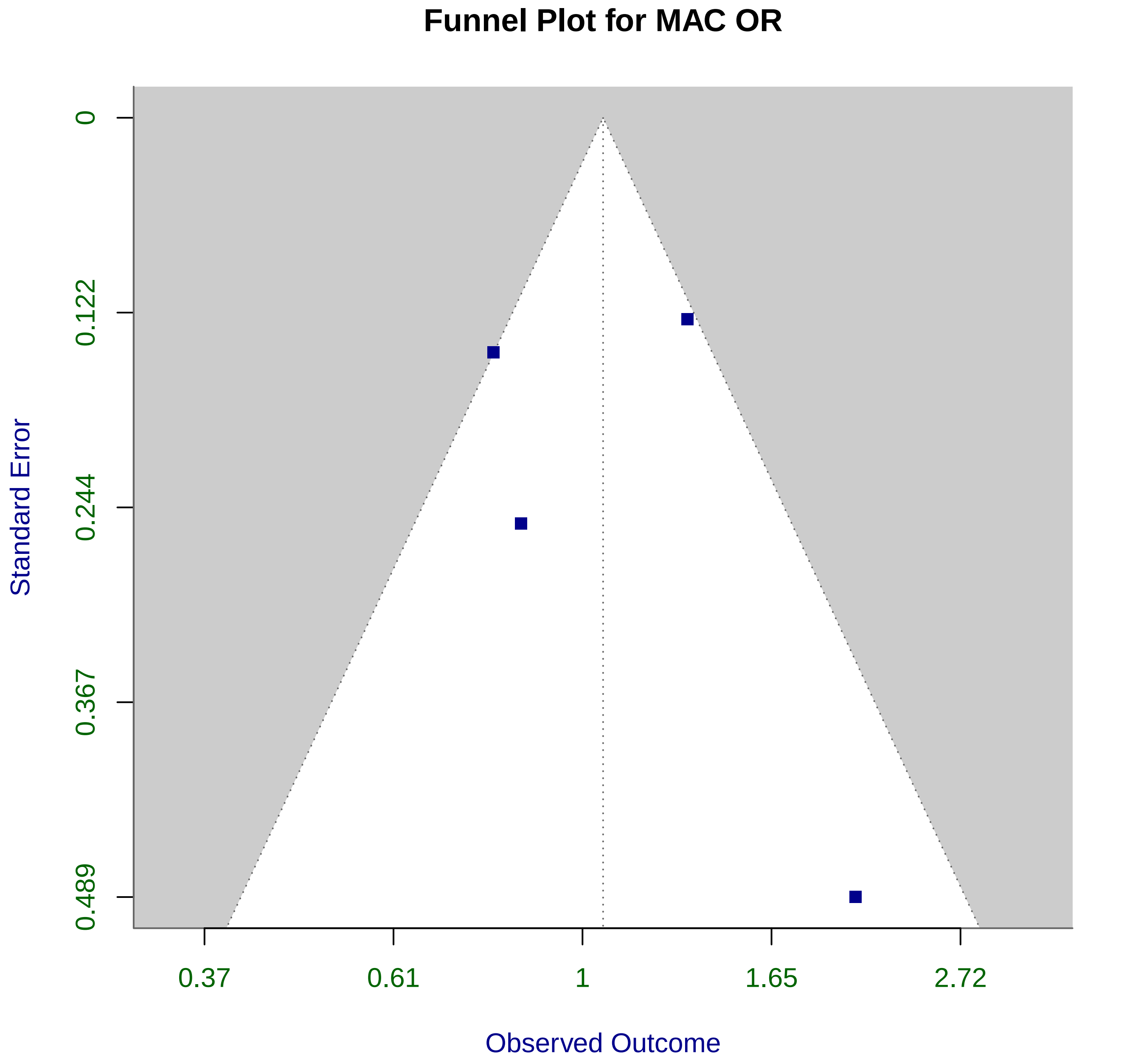

Funnel Plot for MAC OR
Standard Error
0
0.122
0.244
0.367
0.489
0.37
0.61
1
1.65
2.72
Observed Outcome

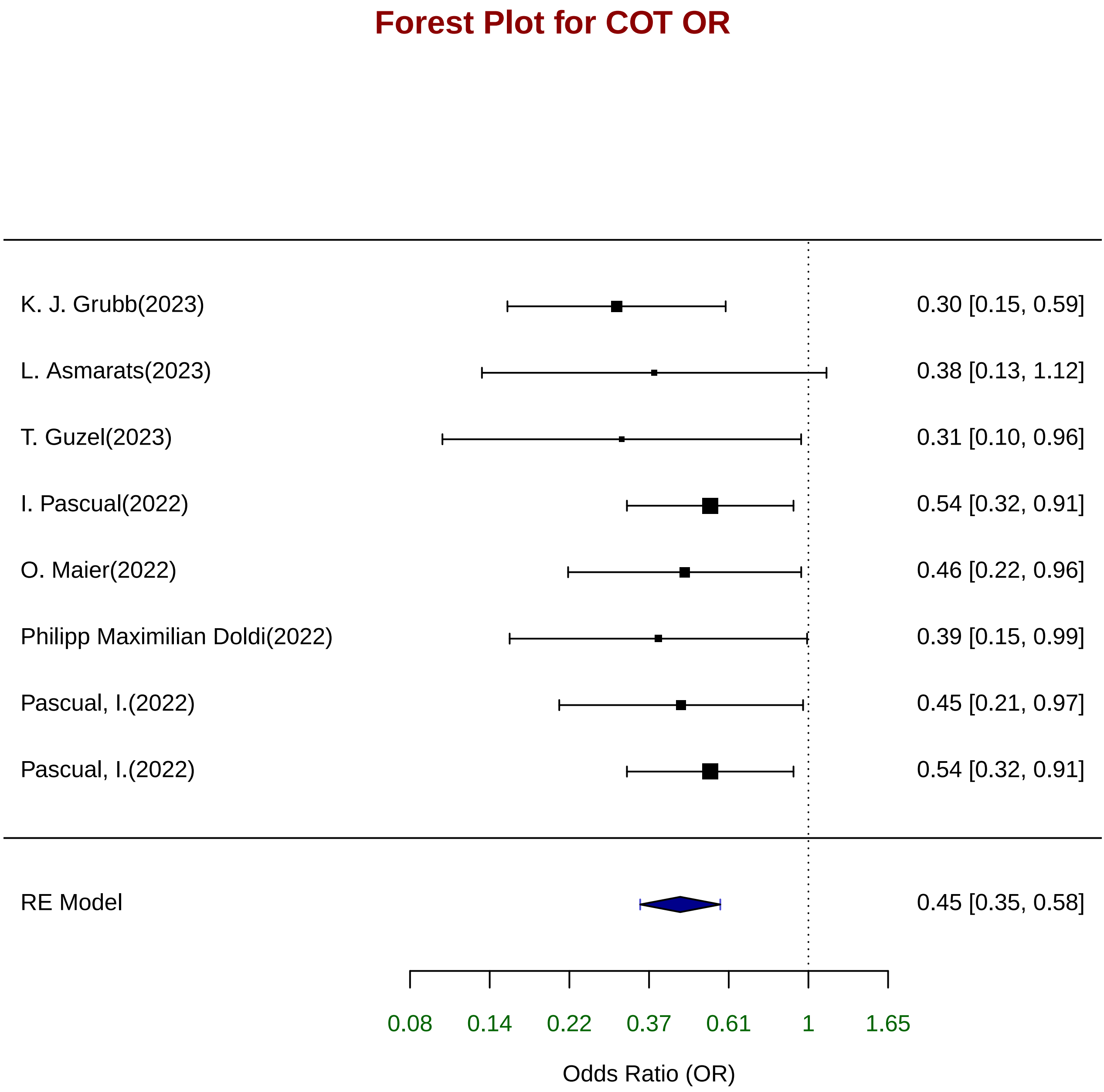
Forest Plot for COT OR
K. J. Grubb(2023)
0.30 [0.15, 0.59]
L. Asmarats(2023)
0.38 [0.13, 1.12]
T. Guzel(2023)
0.31 [0.10, 0.96]
I. Pascual(2022)
0.54 [0.32, 0.91]
O. Maier(2022)
0.46 [0.22, 0.96]
Philipp Maximilian Doldi(2022)
0.39 [0.15, 0.99]
Pascual, I.(2022)
0.45 [0.21, 0.97]
Pascual, I.(2022)
0.54 [0.32, 0.91]
RE Model
0.45 [0.35, 0.58]
0.08
0.14
0.22
0.37
0.61
1
1.65
Odds Ratio (OR)

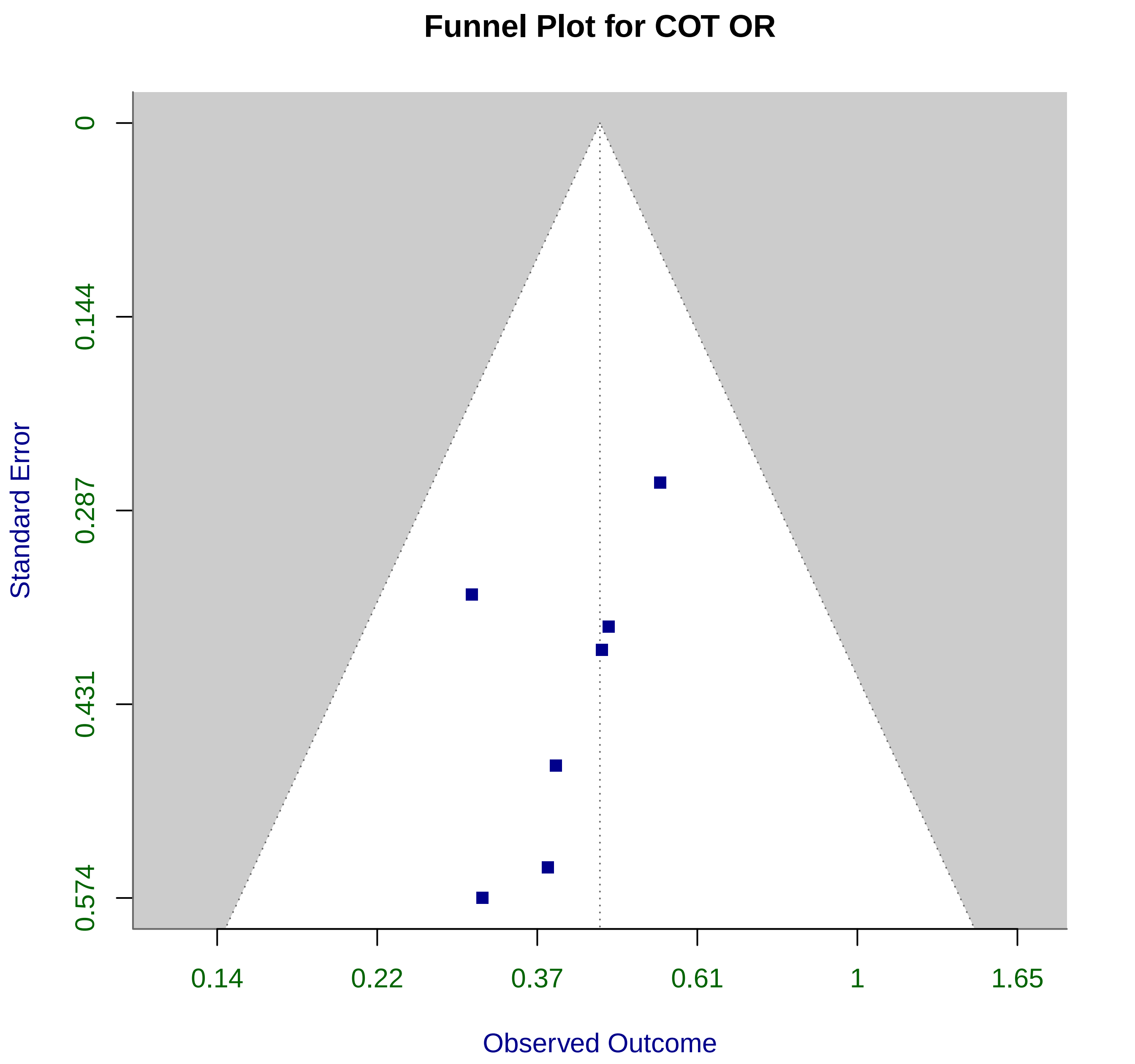
Funnel Plot for COT OR
Standard Error
0
0.144
0.287
0.431
0.574
0.14
0.22
0.37
0.61
1
1.65
Observed Outcome

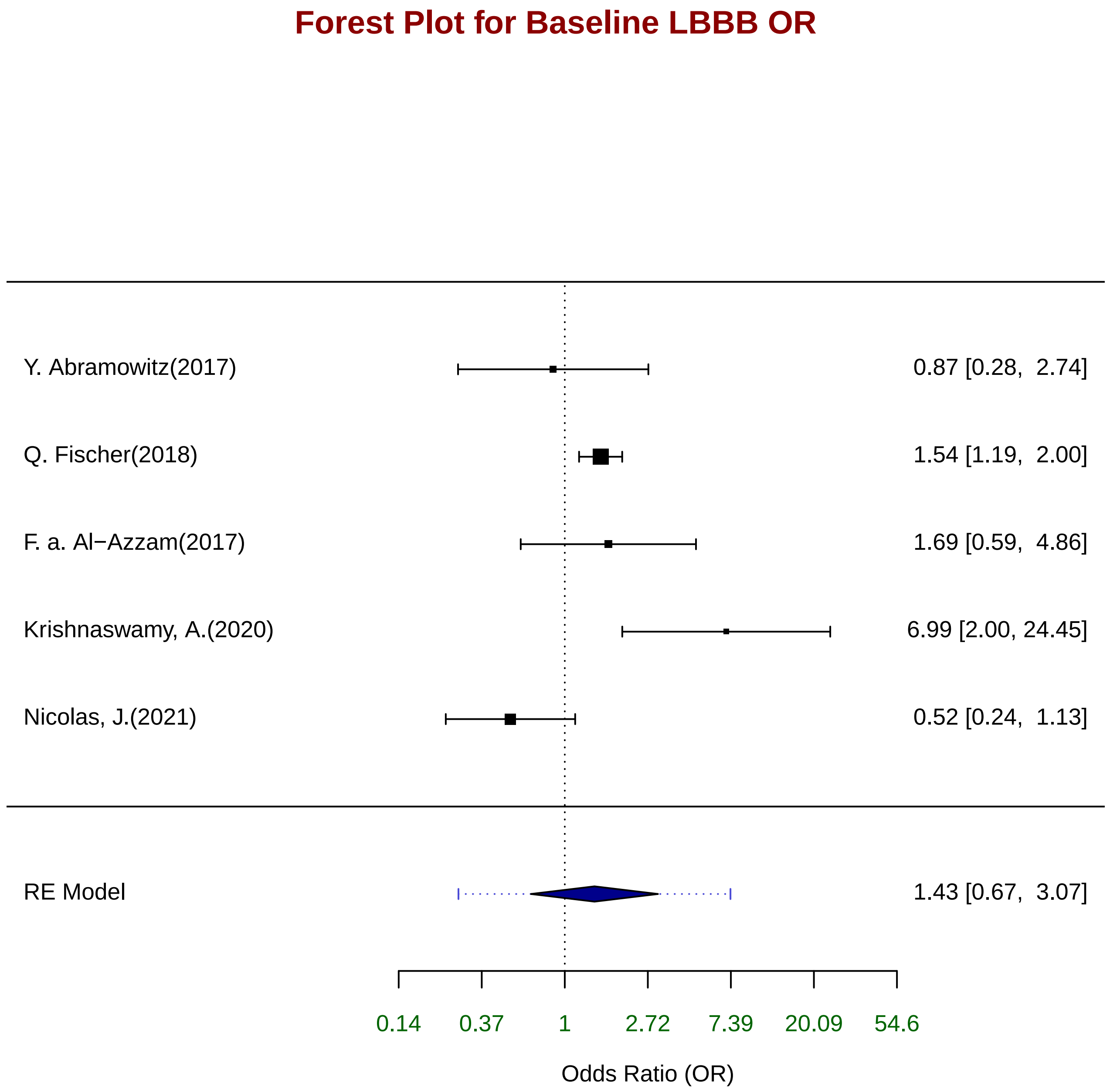

Forest Plot for Baseline LBBB OR
Y. Abramowitz(2017)
0.87 [0.28, 2.74]
Q. Fischer(2018)
1.54 [1.19, 2.00]
F. a. Al−Azzam(2017)
1.69 [0.59, 4.86]
Krishnaswamy, A.(2020)
6.99 [2.00, 24.45]
Nicolas, J.(2021)
0.52 [0.24, 1.13]
RE Model
1.43 [0.67, 3.07]
0.14
0.37
1
2.72
7.39
20.09
54.6
Odds Ratio (OR)

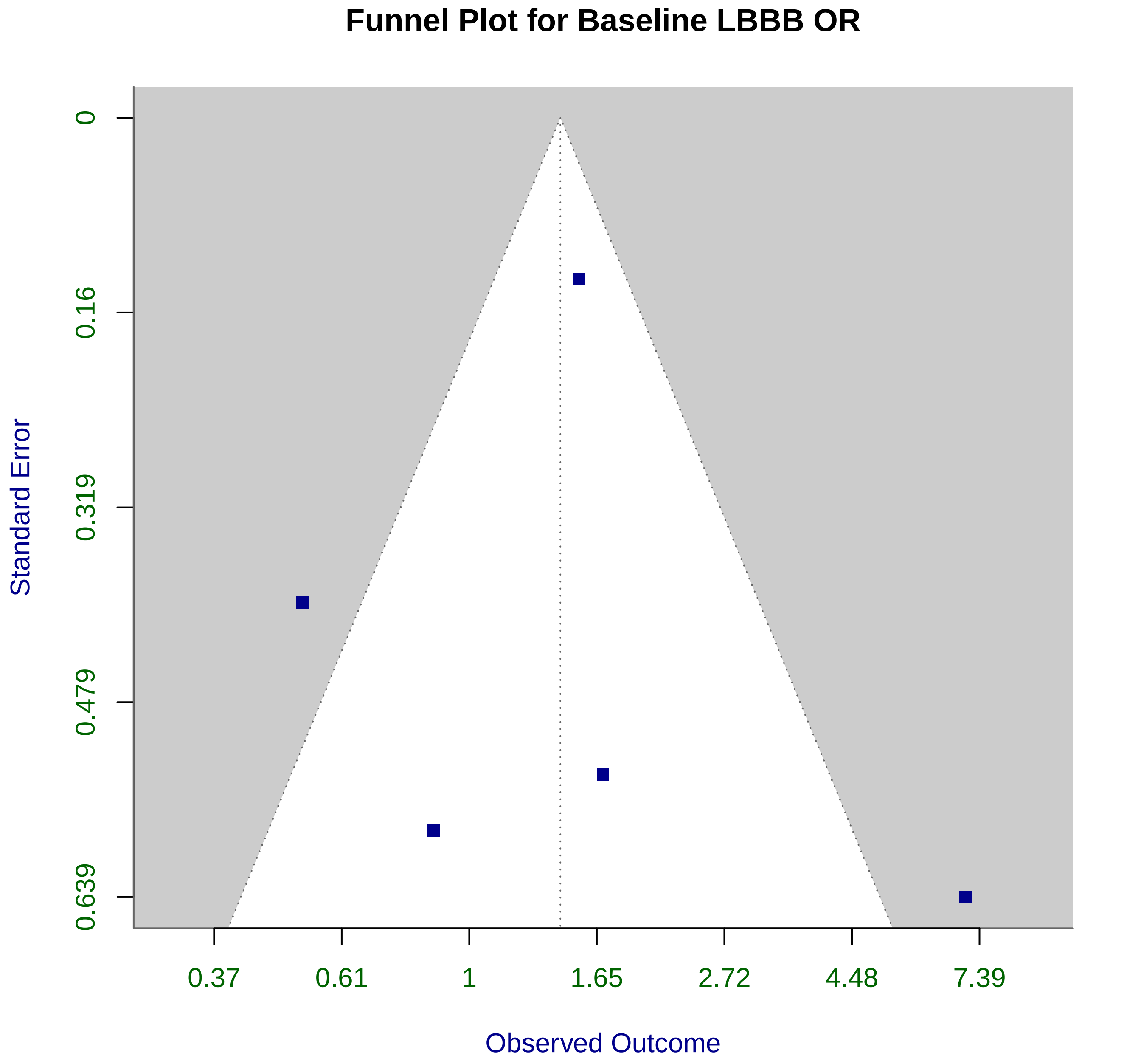
Funnel Plot for Baseline LBBB OR
Standard Error
0
0.16
0.319
0.479
0.639
0.37
0.61
1
1.65
2.72
4.48
7.39
Observed Outcome

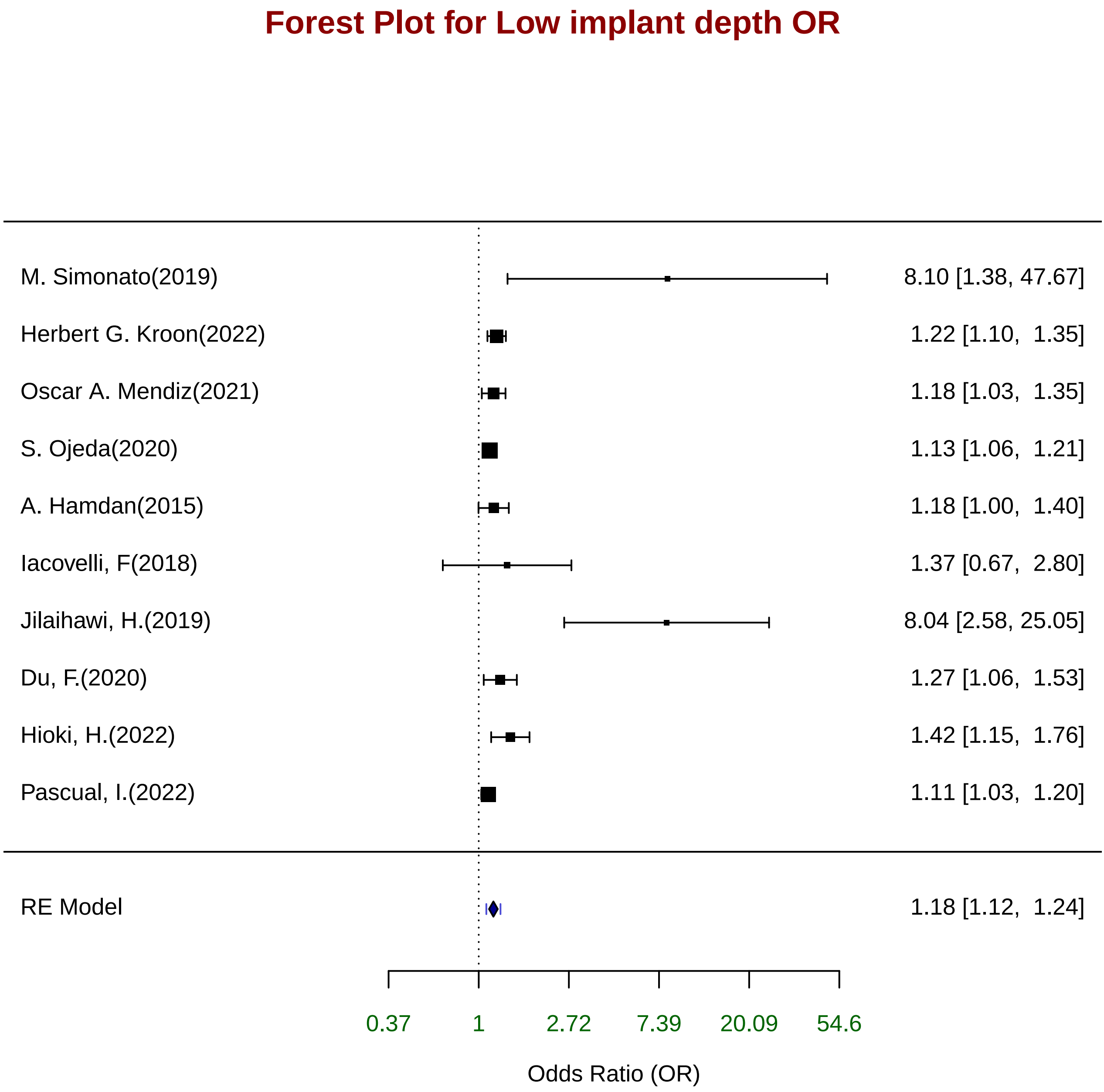
Forest Plot for Low implant depth OR
M. Simonato(2019)
8.10 [1.38, 47.67]
Herbert G. Kroon(2022)
1.22 [1.10, 1.35]
Oscar A. Mendiz(2021)
1.18 [1.03, 1.35]
S. Ojeda(2020)
1.13 [1.06, 1.21]
A. Hamdan(2015)
1.18 [1.00, 1.40]
Iacovelli, F(2018)
1.37 [0.67, 2.80]
Jilaihawi, H.(2019)
8.04 [2.58, 25.05]
Du, F.(2020)
1.27 [1.06, 1.53]
Hioki, H.(2022)
1.42 [1.15, 1.76]
Pascual, I.(2022)
1.11 [1.03, 1.20]
RE Model
1.18 [1.12, 1.24]
0.37
1
2.72
7.39
20.09
54.6
Odds Ratio (OR)

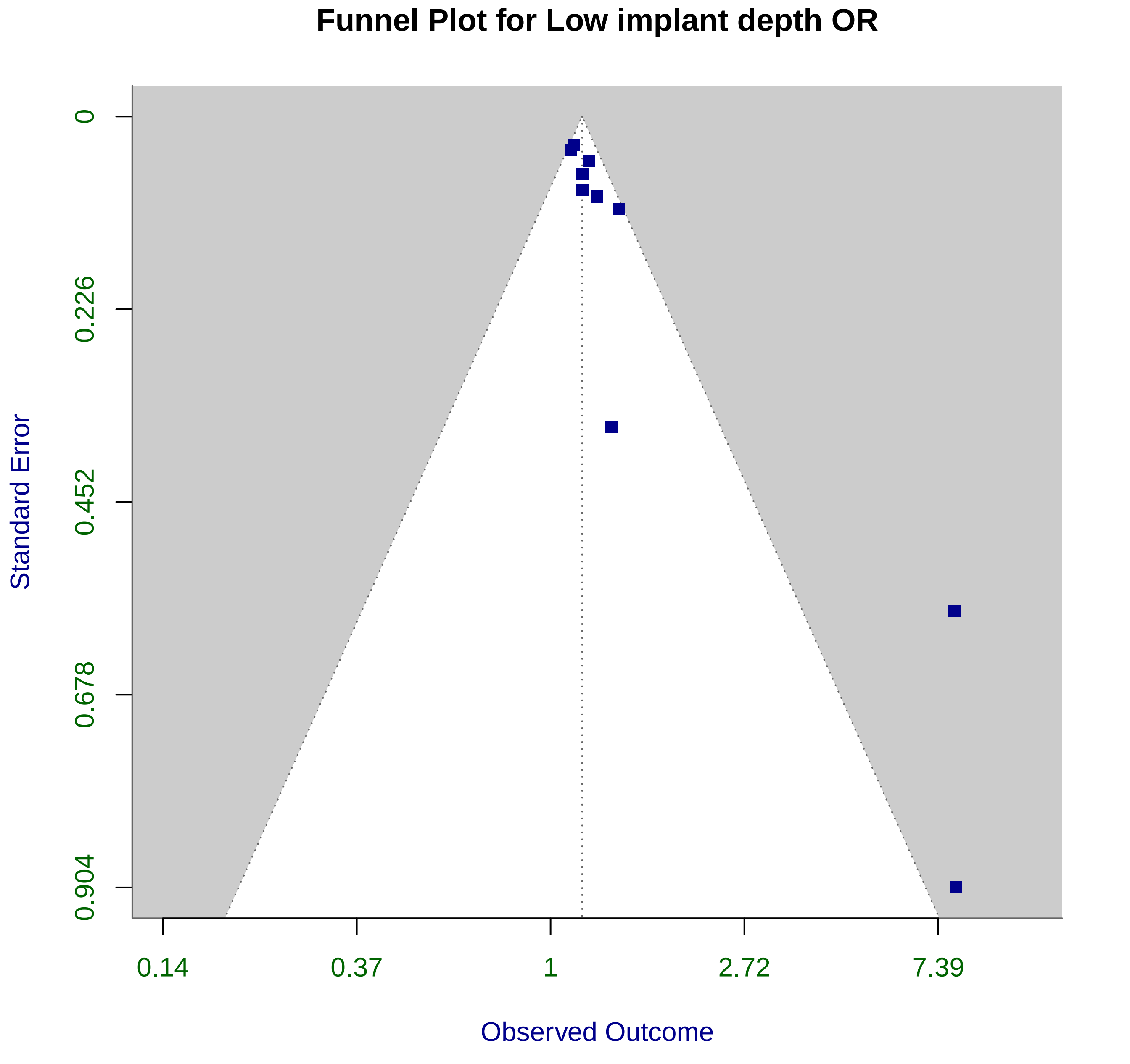

Funnel Plot for Low implant depth OR
Standard Error
0
0.226
0.452
0.678
0.904
0.14
0.37
1
2.72
7.39
Observed Outcome

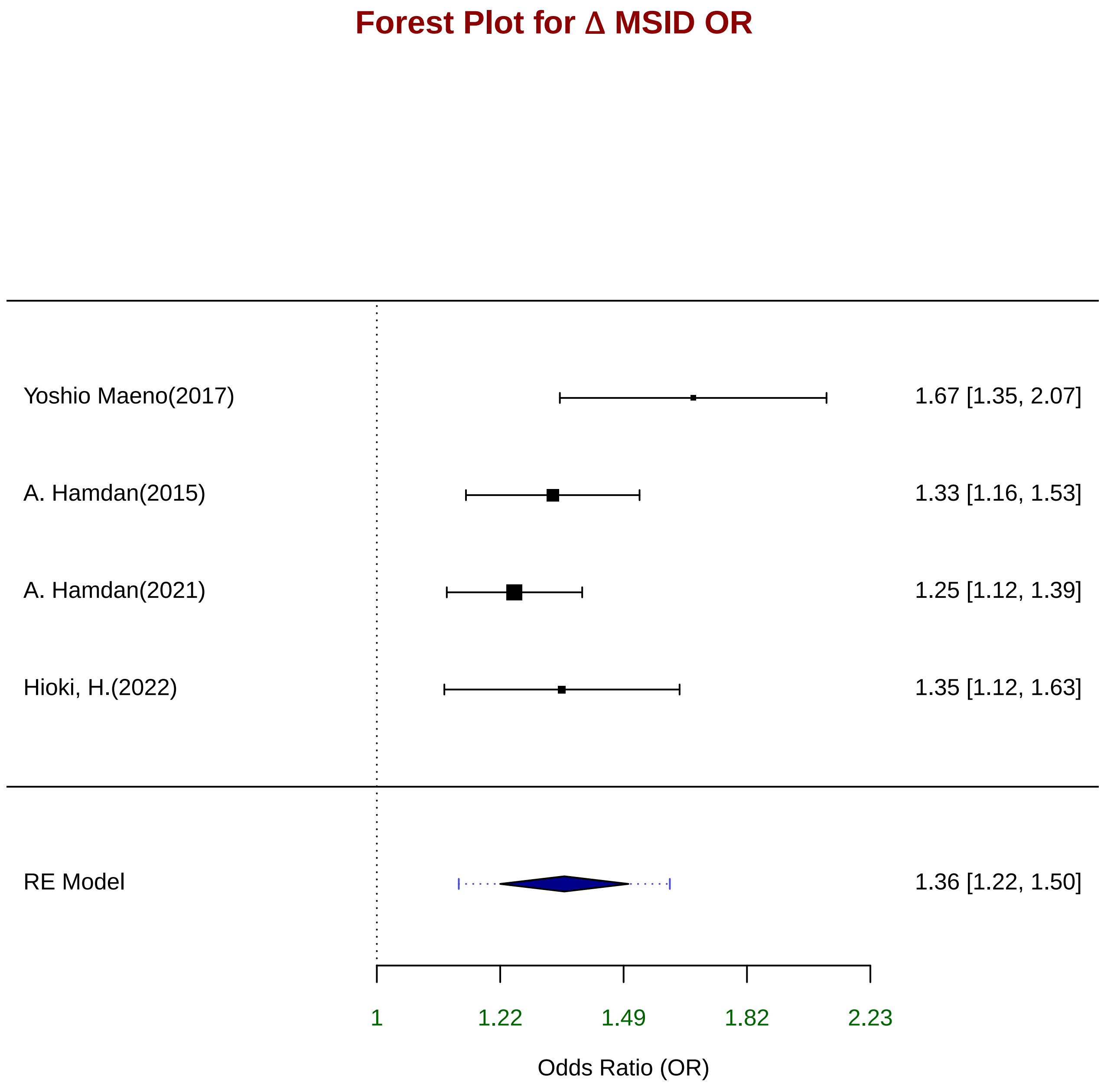
Forest Plot for Δ MSID OR
Yoshio Maeno(2017)
1.67 [1.35, 2.07]
A. Hamdan(2015)
1.33 [1.16, 1.53]
A. Hamdan(2021)
1.25 [1.12, 1.39]
Hioki, H.(2022)
1.35 [1.12, 1.63]
RE Model
1.36 [1.22, 1.50]
1
1.22
1.49
1.82
2.23
Odds Ratio (OR)

Funnel Plot for Δ MSID OR

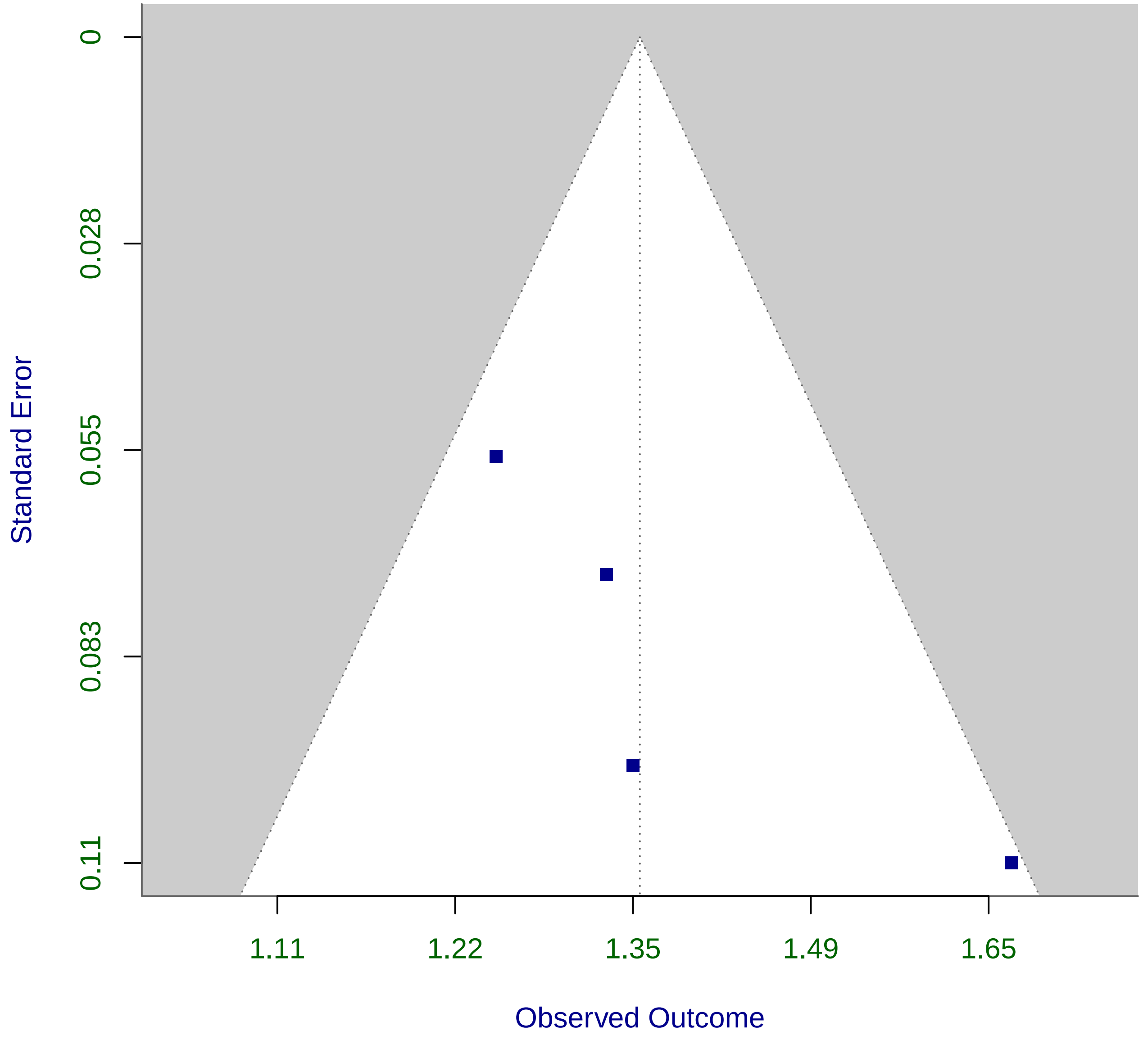

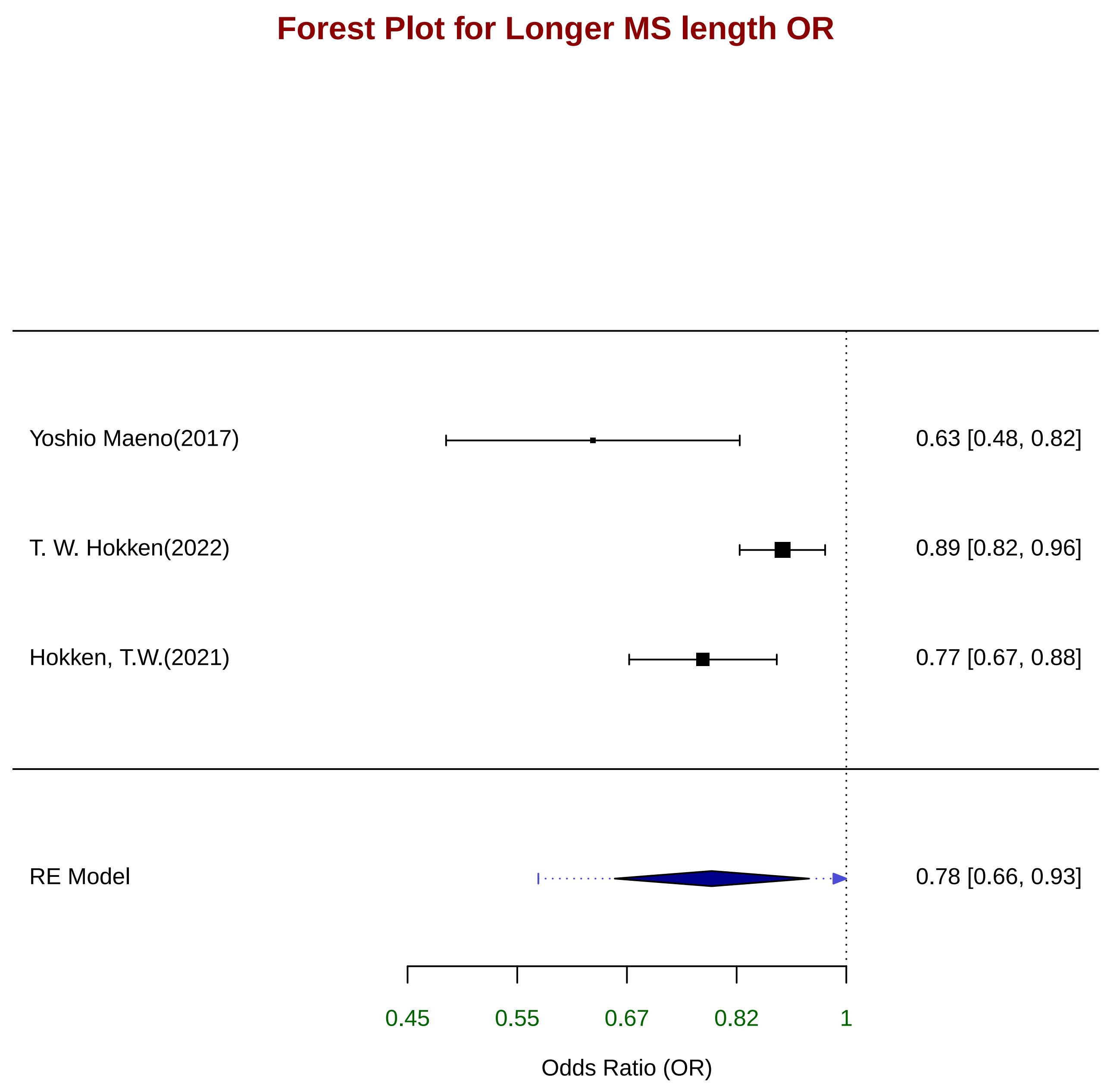
Forest Plot for Longer MS length OR
Yoshio Maeno(2017)
0.63 [0.48, 0.82]
T. W. Hokken(2022)
0.89 [0.82, 0.96]
Hokken, T.W.(2021)
0.77 [0.67, 0.88]
RE Model
0.78 [0.66, 0.93]
0.45
0.55
0.67
0.82
1
Odds Ratio (OR)

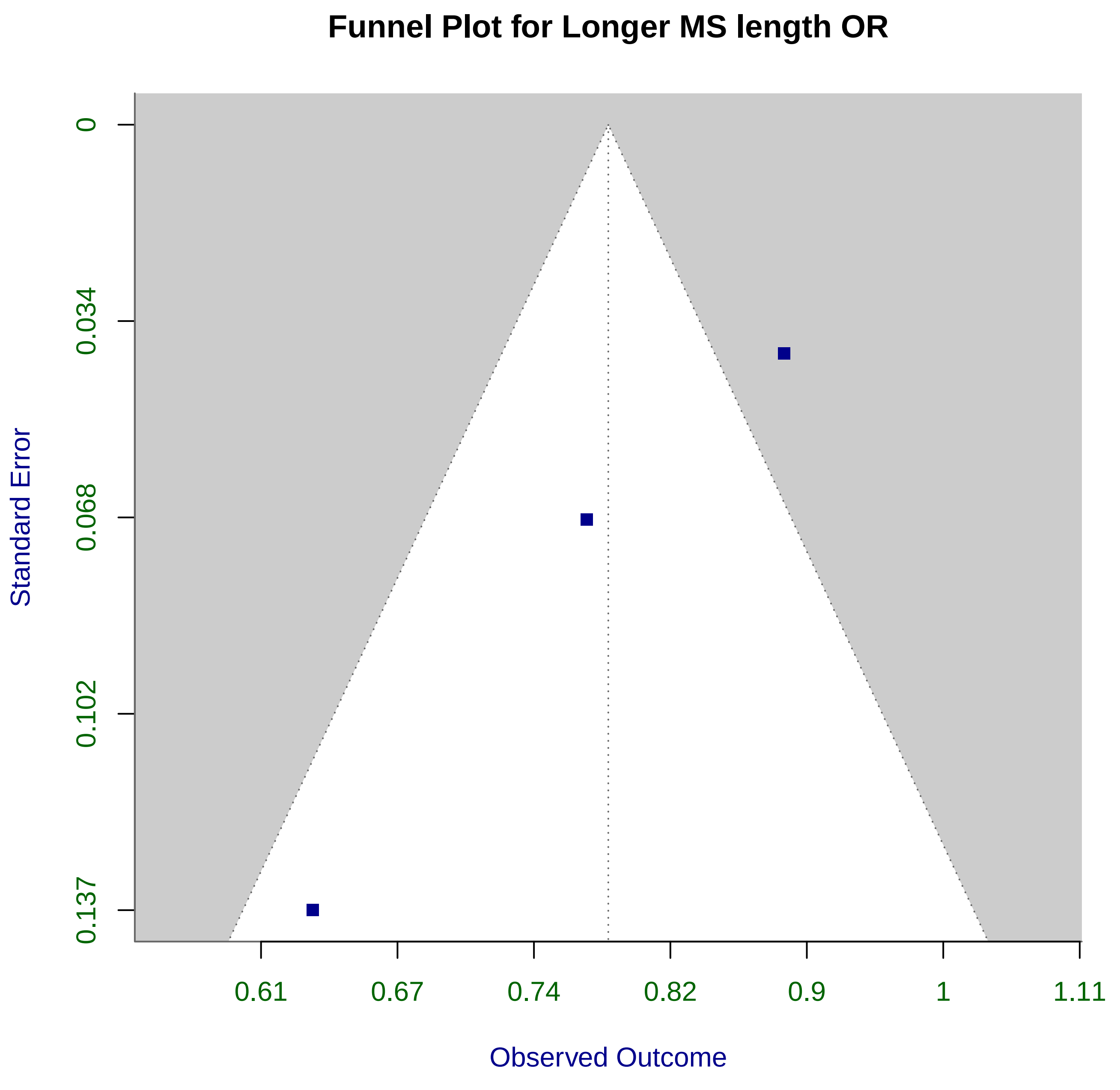
Funnel Plot for Longer MS length OR
Standard Error
0
0.034
0.068
0.102
0.137
Observed Outcome
0.61
0.67
0.74
0.82
0.9
1
1.11

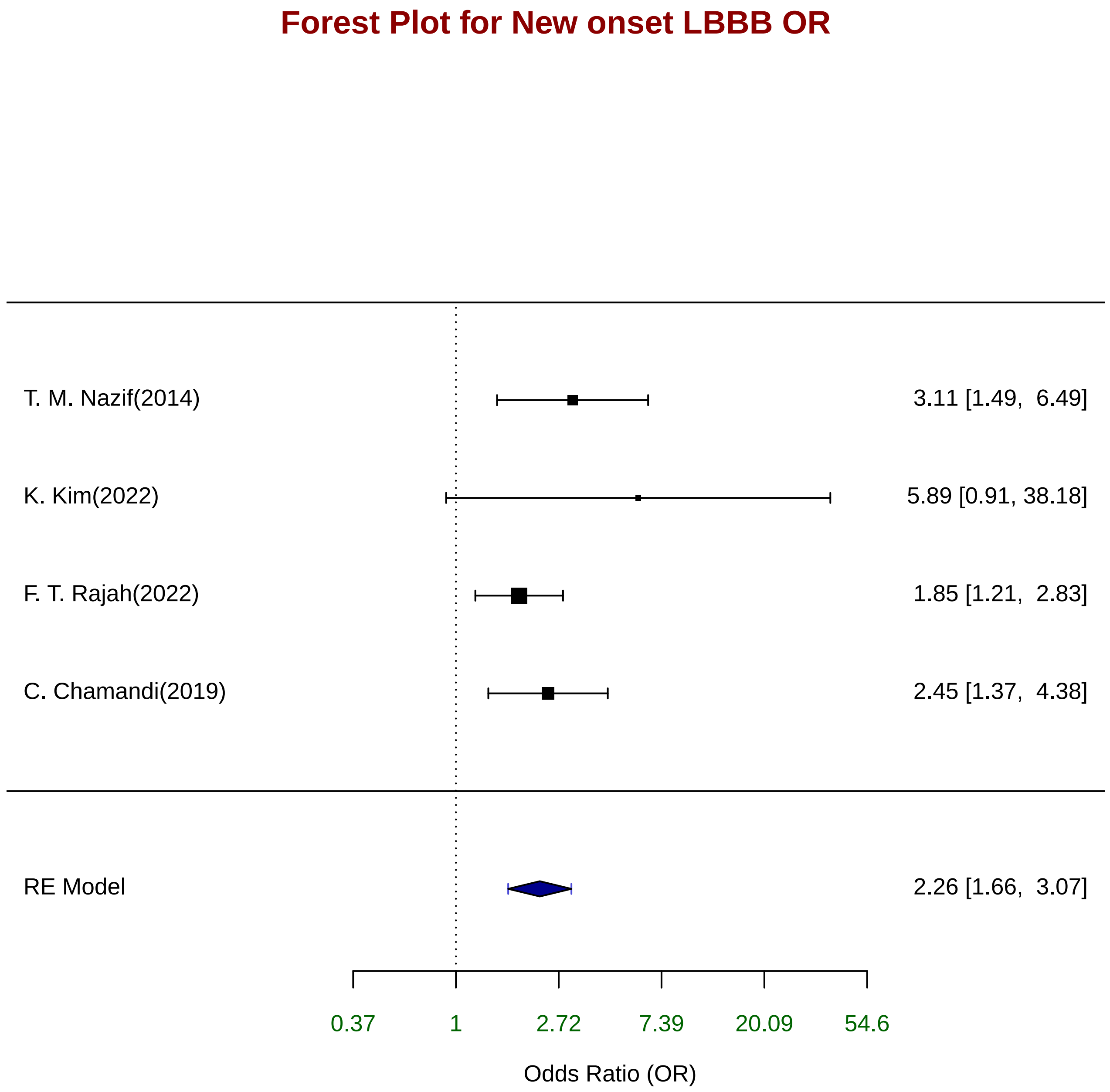
Forest Plot for New onset LBBB OR
T. M. Nazif(2014)
3.11 [1.49, 6.49]
K. Kim(2022)
5.89 [0.91, 38.18]
F. T. Rajah(2022)
1.85 [1.21, 2.83]
C. Chamandi(2019)
2.45 [1.37, 4.38]
RE Model
2.26 [1.66, 3.07]
0.37
1
2.72
7.39
20.09
54.6
Odds Ratio (OR)

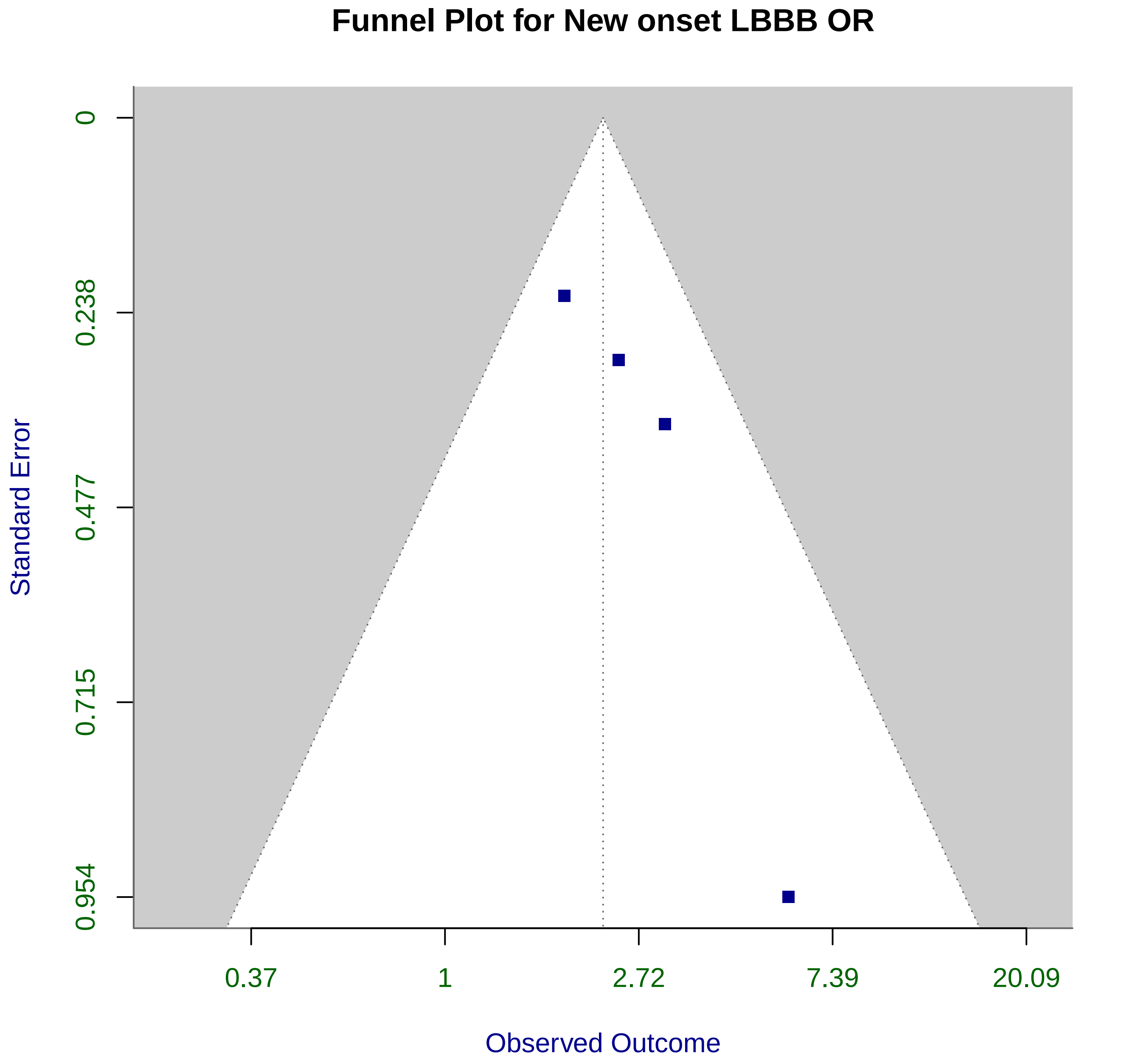
Funnel Plot for New onset LBBB OR
Standard Error
0
0.238
0.477
0.715
0.954
0.37
1
2.72
7.39
20.09
Observed Outcome

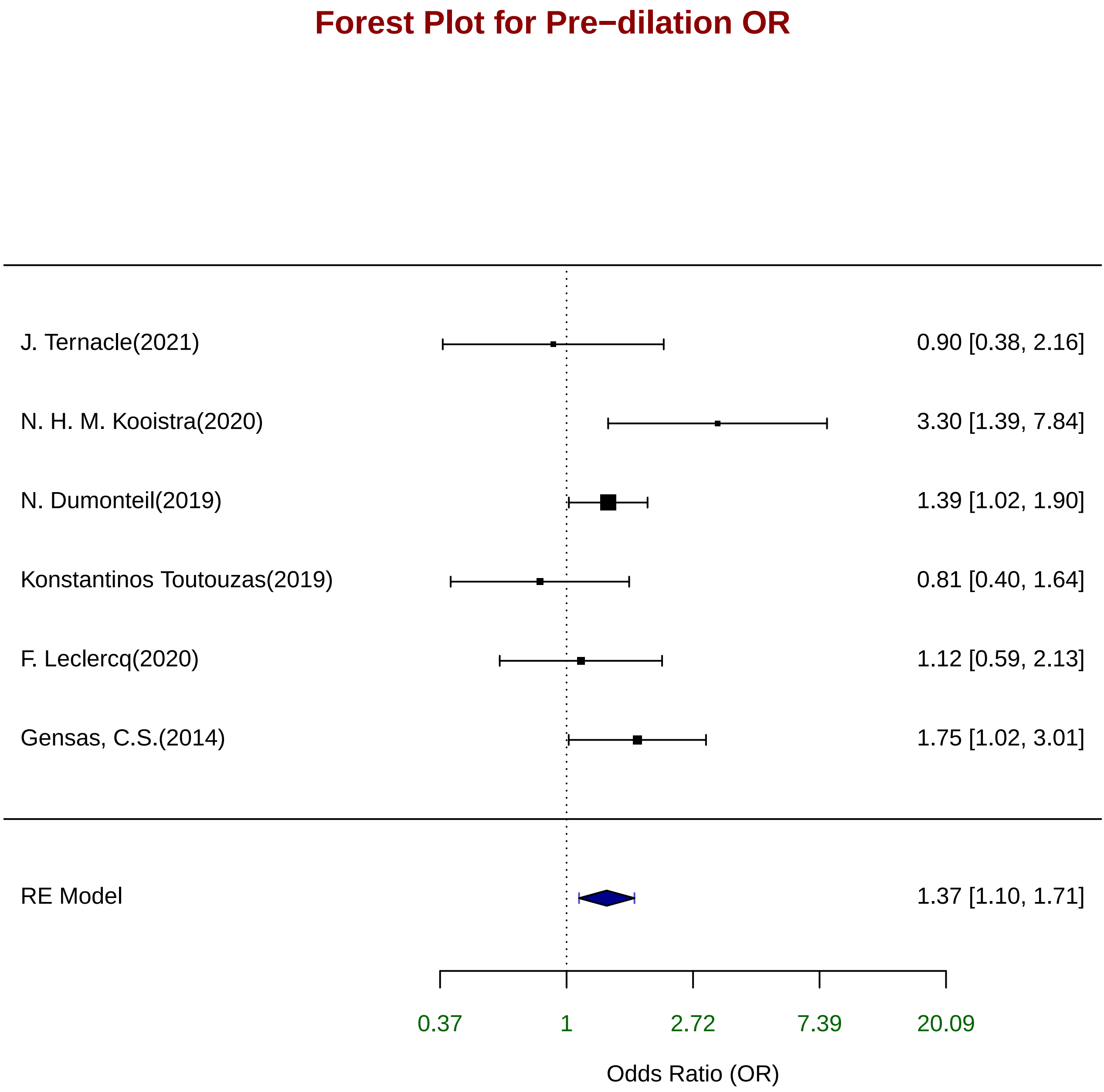

Forest Plot for Pre−dilation OR
J. Ternacle(2021)
0.90 [0.38, 2.16]
N. H. M. Kooistra(2020)
3.30 [1.39, 7.84]
N. Dumonteil(2019)
1.39 [1.02, 1.90]
Konstantinos Toutouzas(2019)
0.81 [0.40, 1.64]
F. Leclercq(2020)
1.12 [0.59, 2.13]
Gensas, C.S.(2014)
1.75 [1.02, 3.01]
RE Model
1.37 [1.10, 1.71]
0.37
1
2.72
7.39
20.09
Odds Ratio (OR)

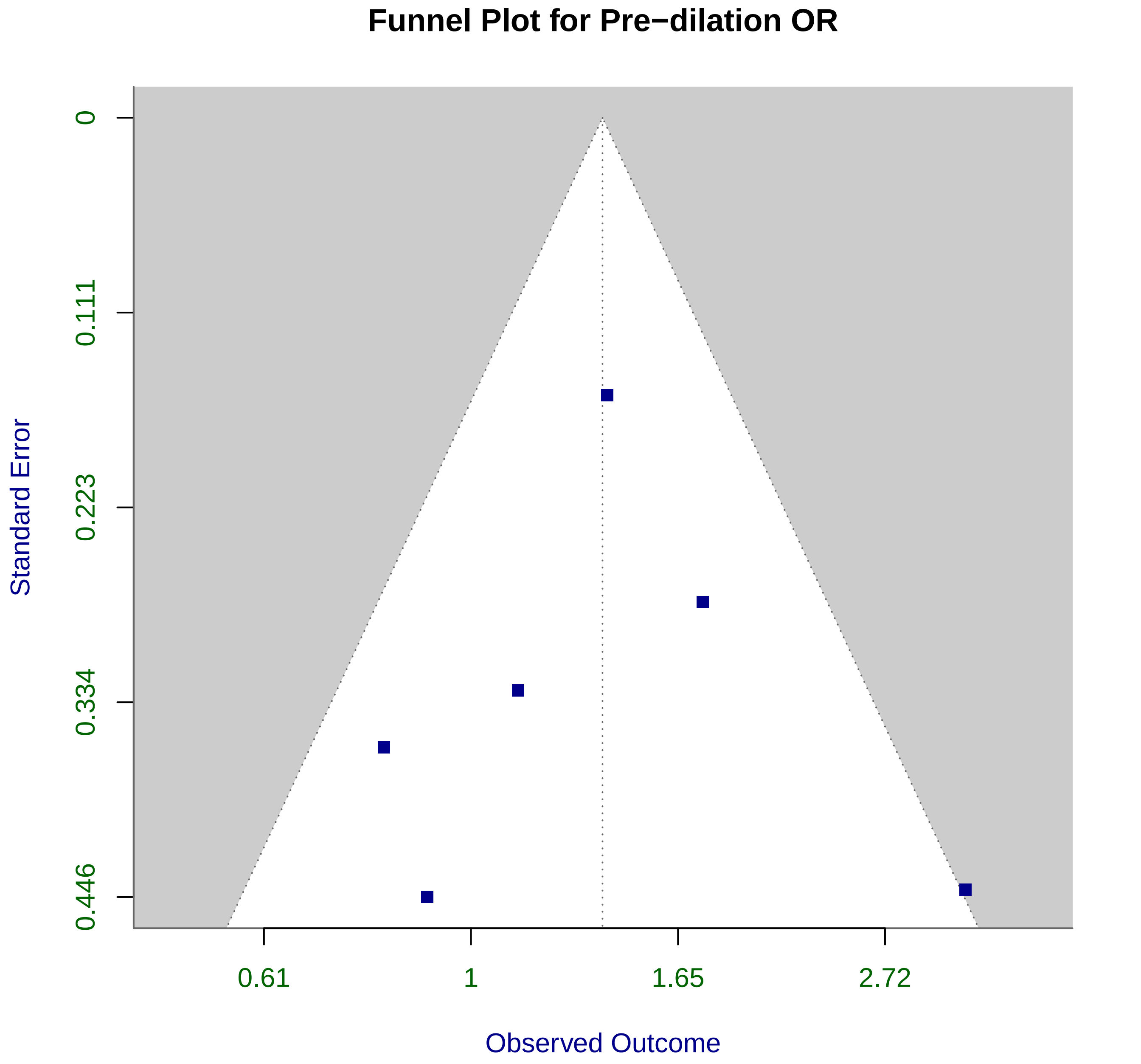

Funnel Plot for Pre−dilation OR
Standard Error
0
0.111
0.223
0.334
0.446
0.61
1
1.65
2.72
Observed Outcome

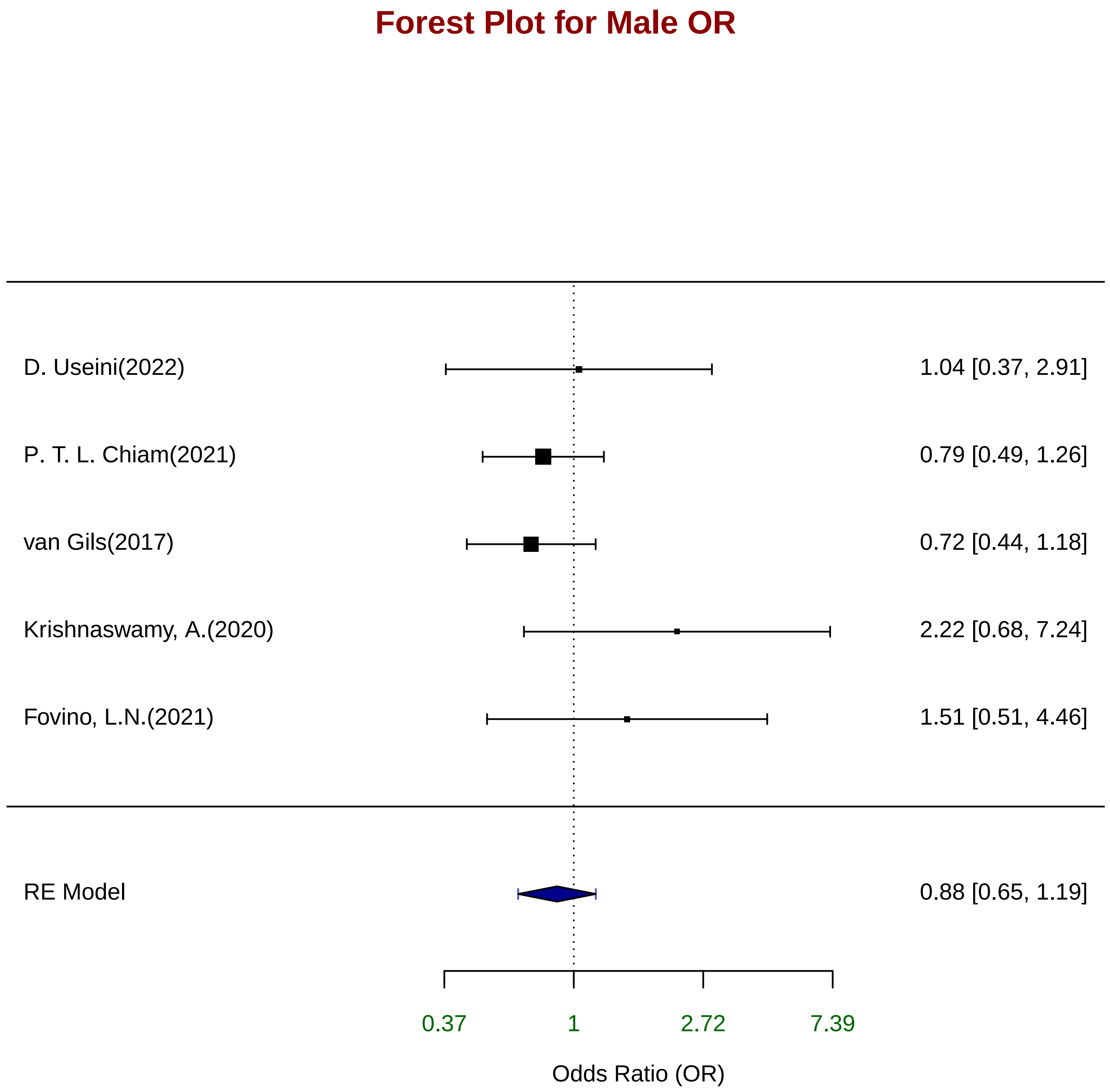
Forest Plot for Male OR
D. Useini(2022)
1.04 [0.37, 2.91]
P. T. L. Chiam(2021)
0.79 [0.49, 1.26]
van Gils(2017)
0.72 [0.44, 1.18]
Krishnaswamy, A.(2020)
2.22 [0.68, 7.24]
Fovino, L.N.(2021)
1.51 [0.51, 4.46]
RE Model
0.88 [0.65, 1.19]
0.37
1
2.72
7.39
Odds Ratio (OR)

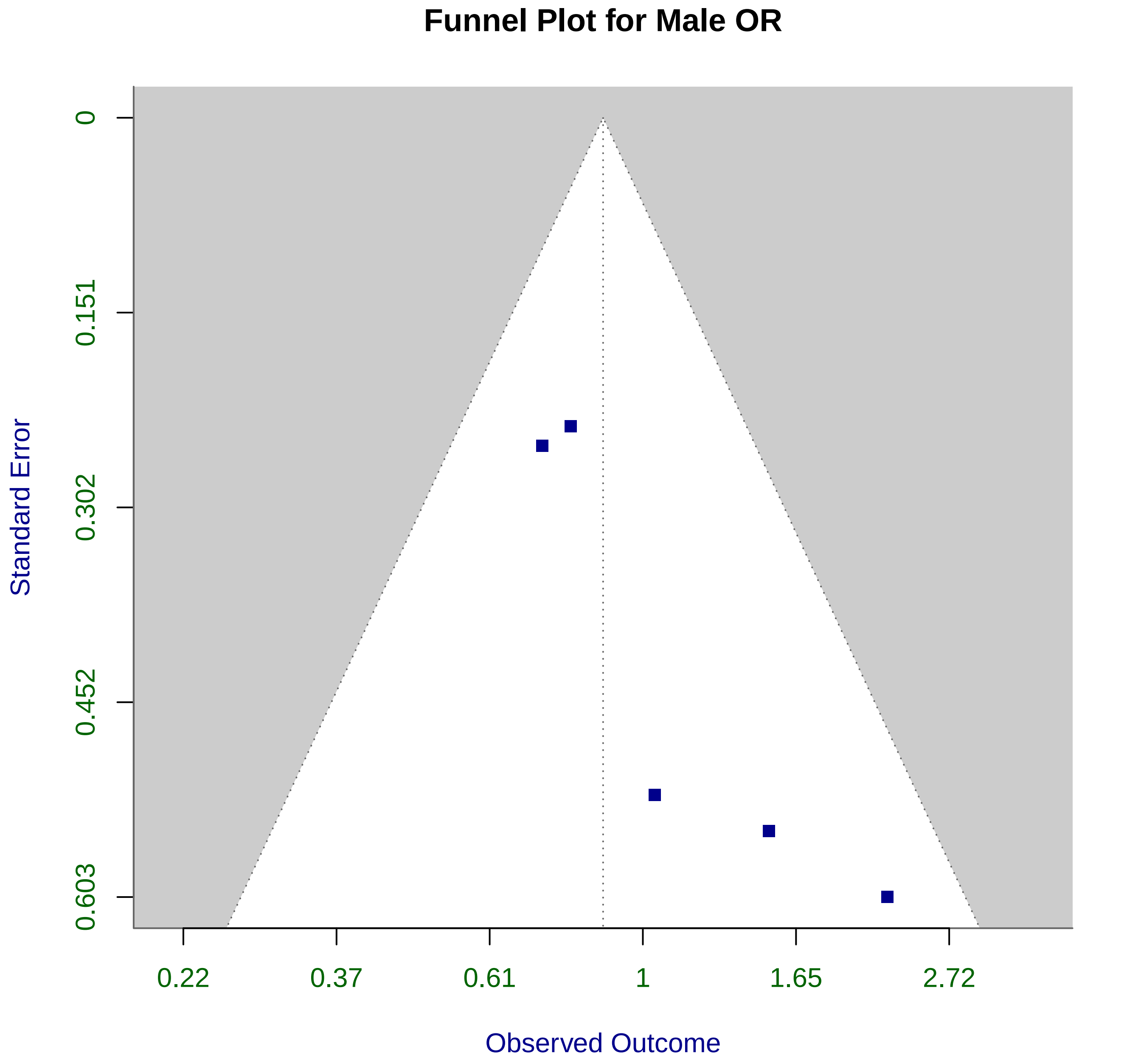

Funnel Plot for Male OR
Standard Error
0
0.151
0.302
0.452
0.603
0.22
0.37
0.61
1
1.65
2.72
Observed Outcome

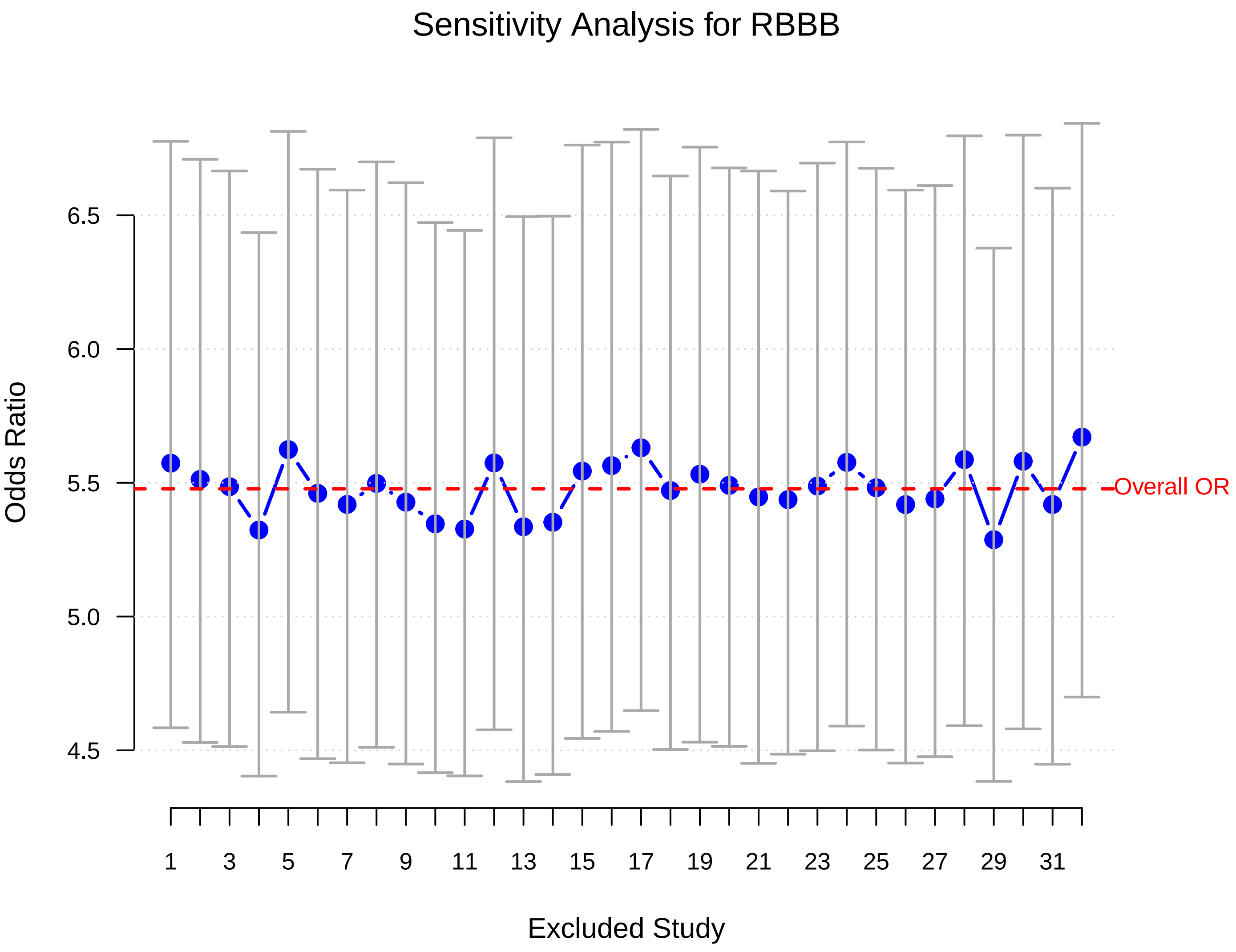
Sensitivity Analysis for RBBB
Odds Ratio
6.5
6.0
5.5
5.0
4.5
Overall OR
1
3
5
7
9
11
13
15
17
19
21
23
25
27
29
31
Excluded Study

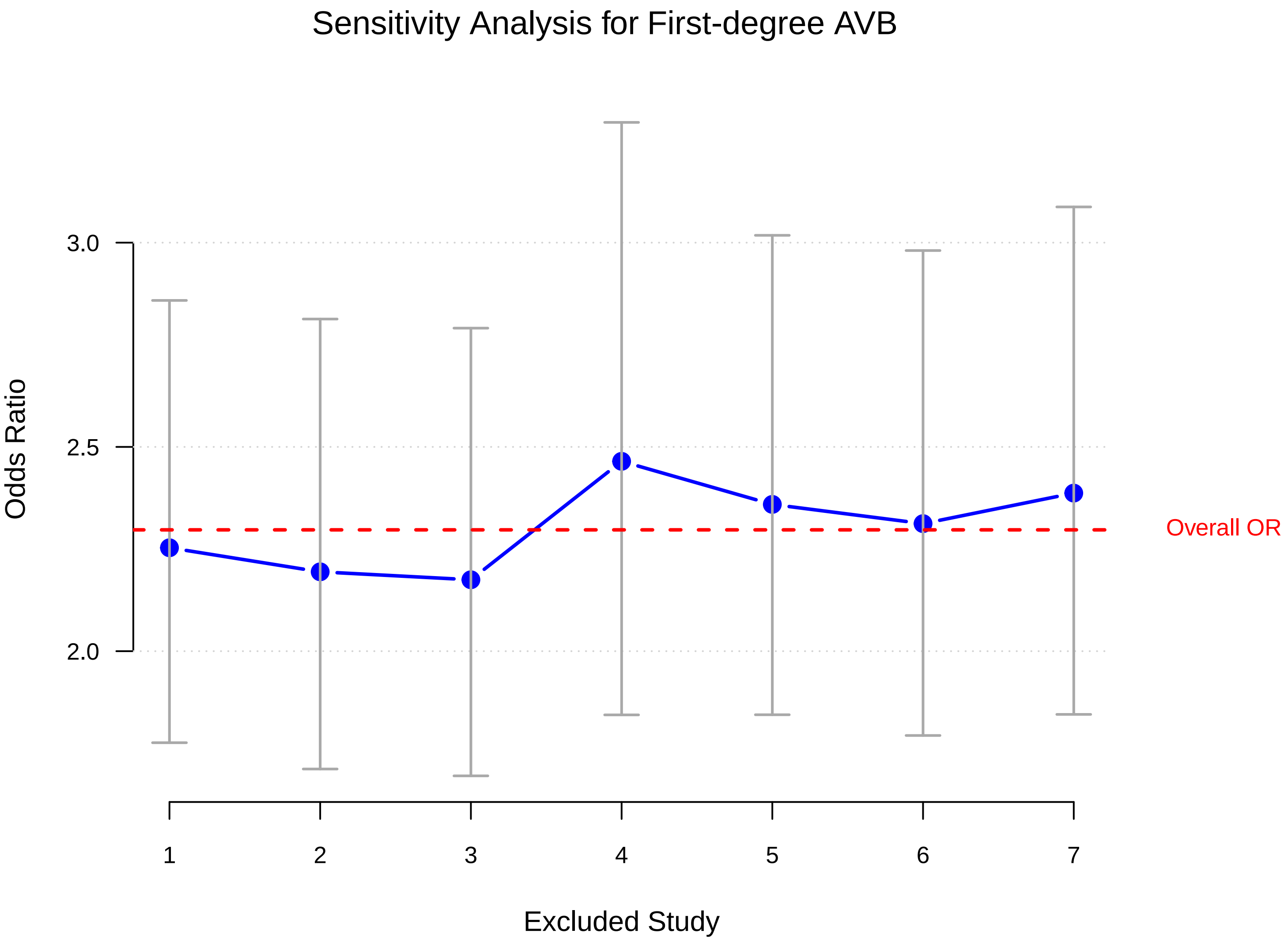
Sensitivity Analysis for First-degree AVB
Odds Ratio
3.0
2.5
2.0
Overall OR
1
2
3
4
5
6
7
Excluded Study

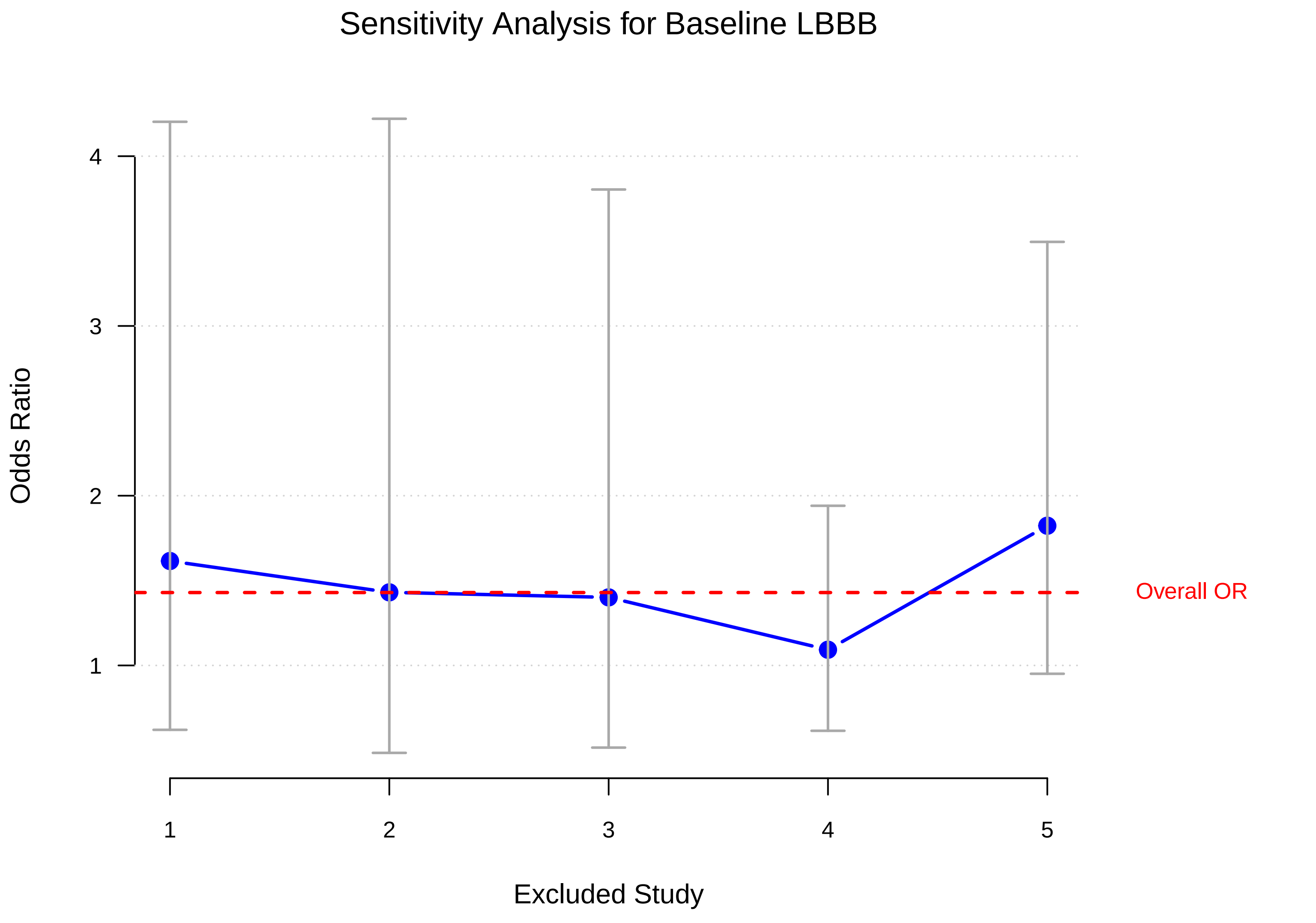

Sensitivity Analysis for Baseline LBBB
Odds Ratio
4
3
2
1
Overall OR
1
2
3
4
5
Excluded Study

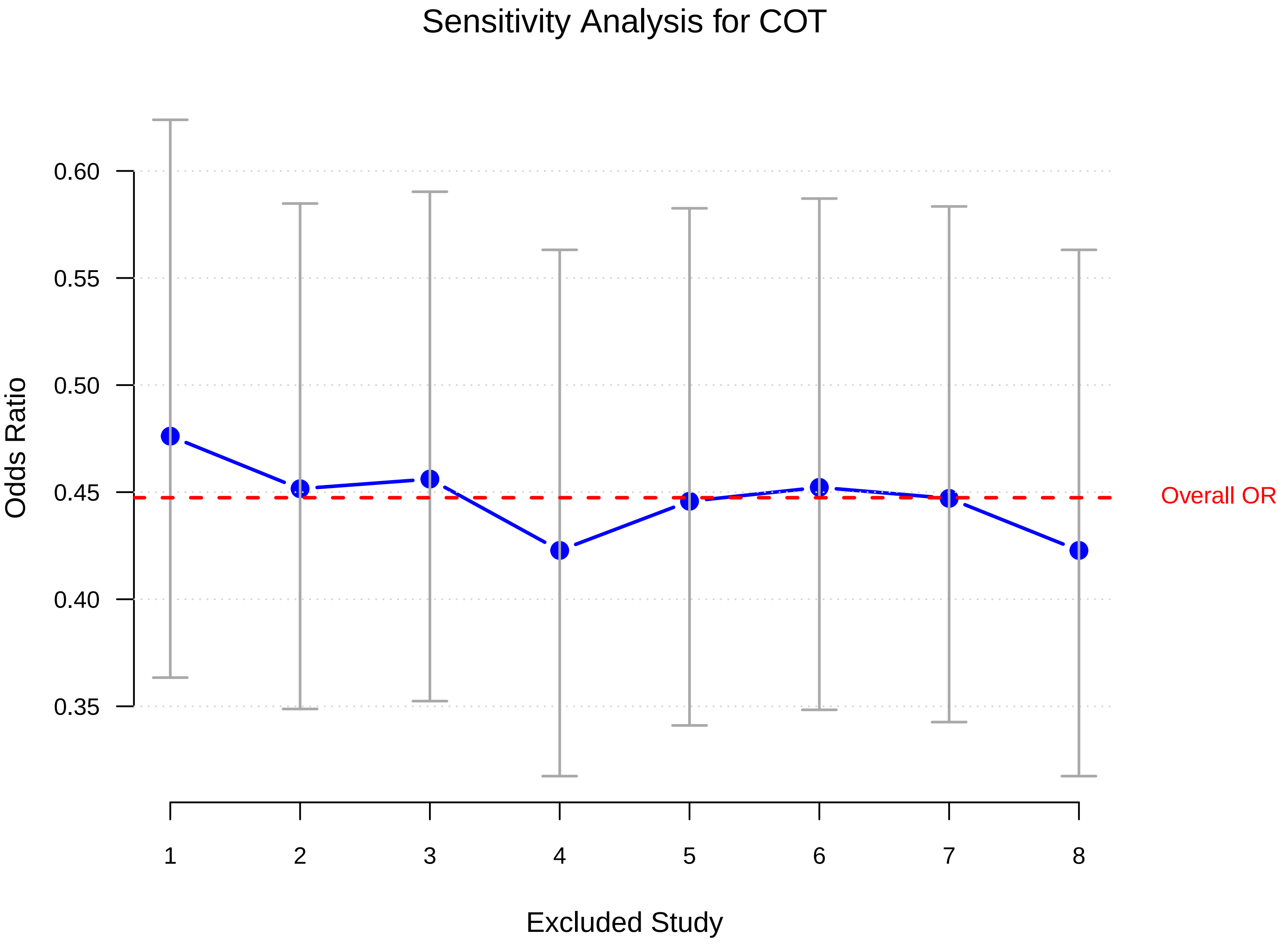
Sensitivity Analysis for COT
Odds Ratio
0.60
0.55
0.50
0.45
0.40
0.35
Overall OR
1
2
3
4
5
6
7
8
Excluded Study

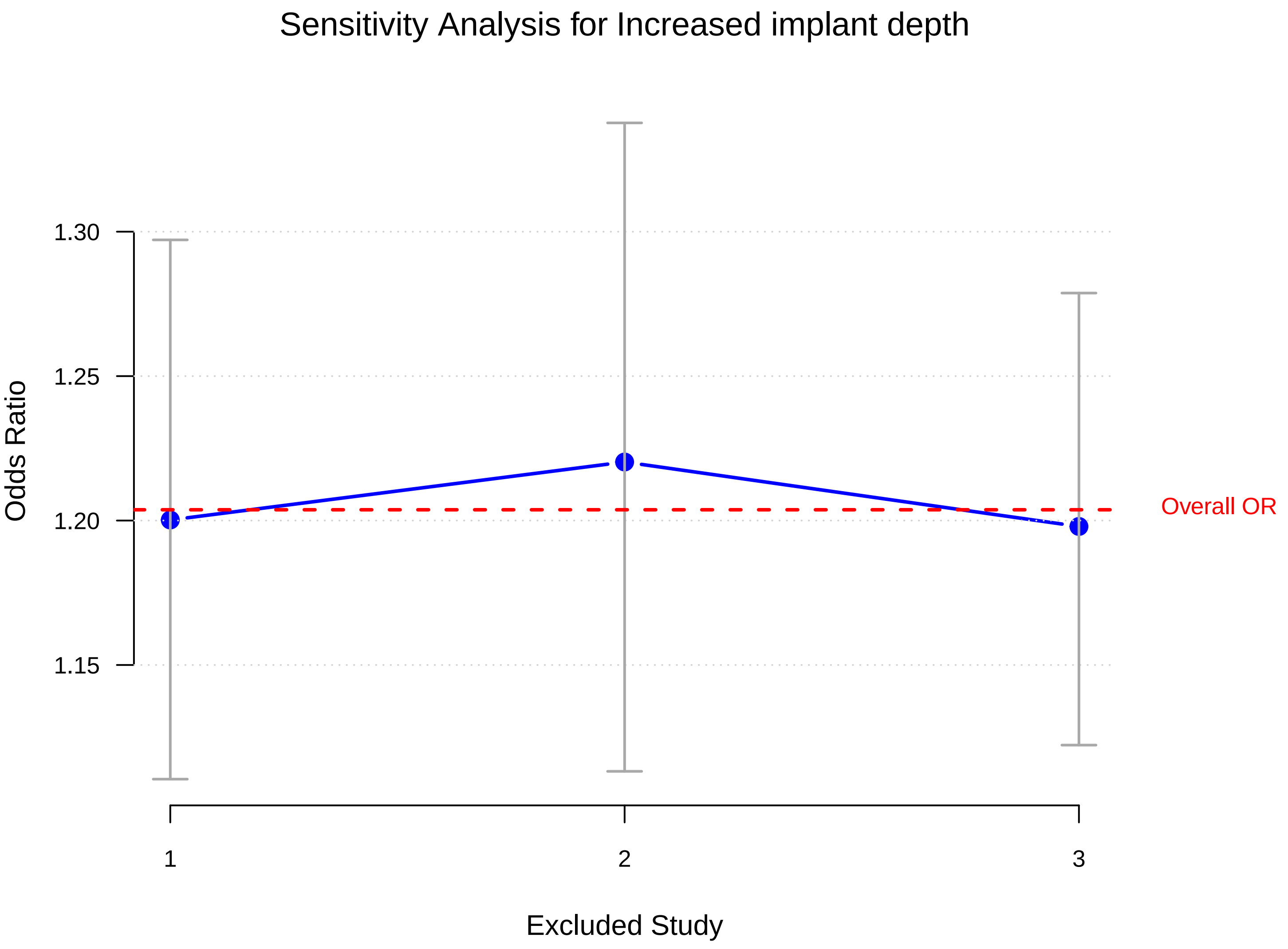

Sensitivity Analysis for Increased implant depth
Odds Ratio
1.30
1.25
1.20
1.15
Overall OR
1
2
3
Excluded Study

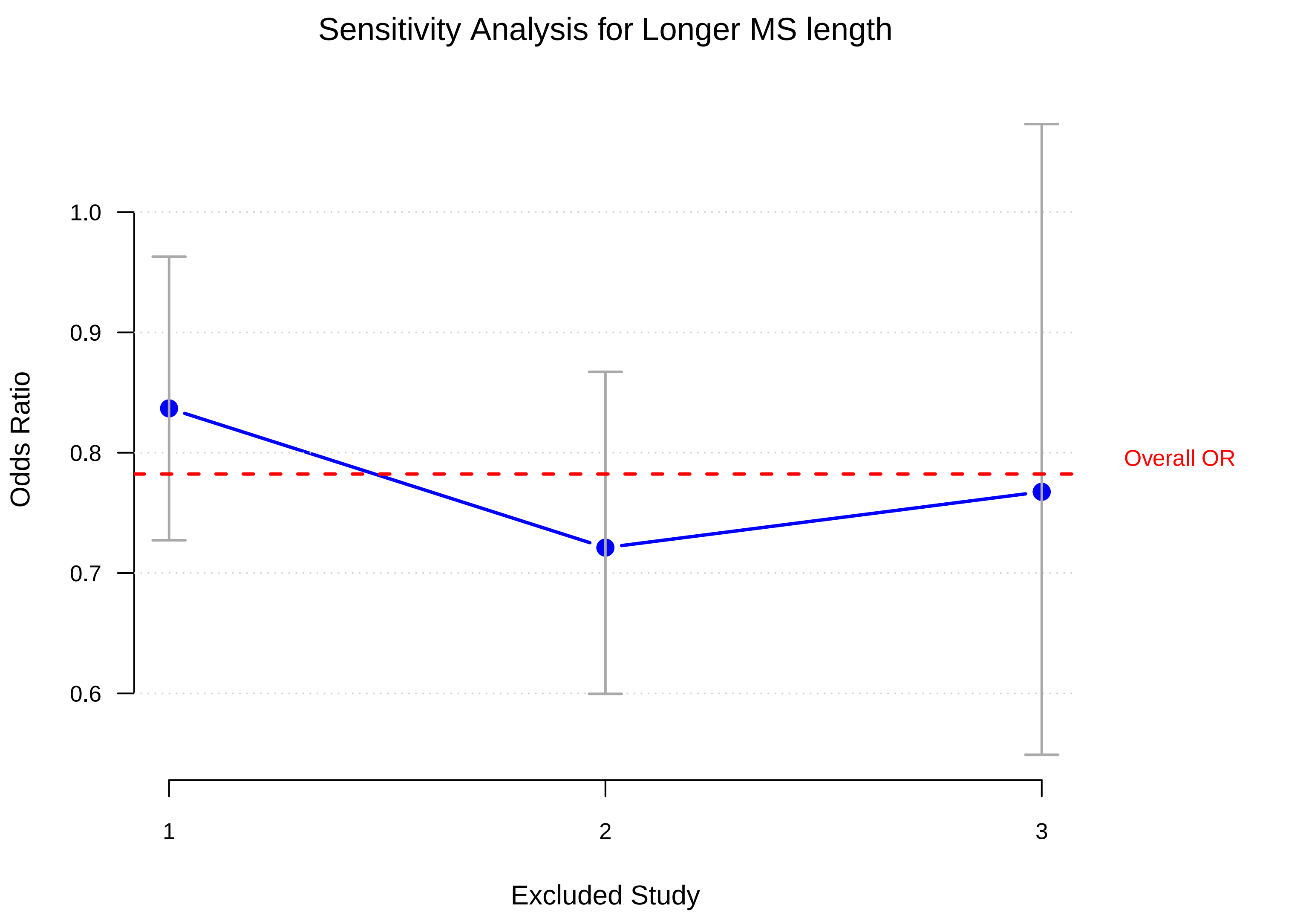
Sensitivity Analysis for Longer MS length
Odds Ratio
1.0
0.9
0.8
0.7
0.6
Overall OR
1
2
3
Excluded Study

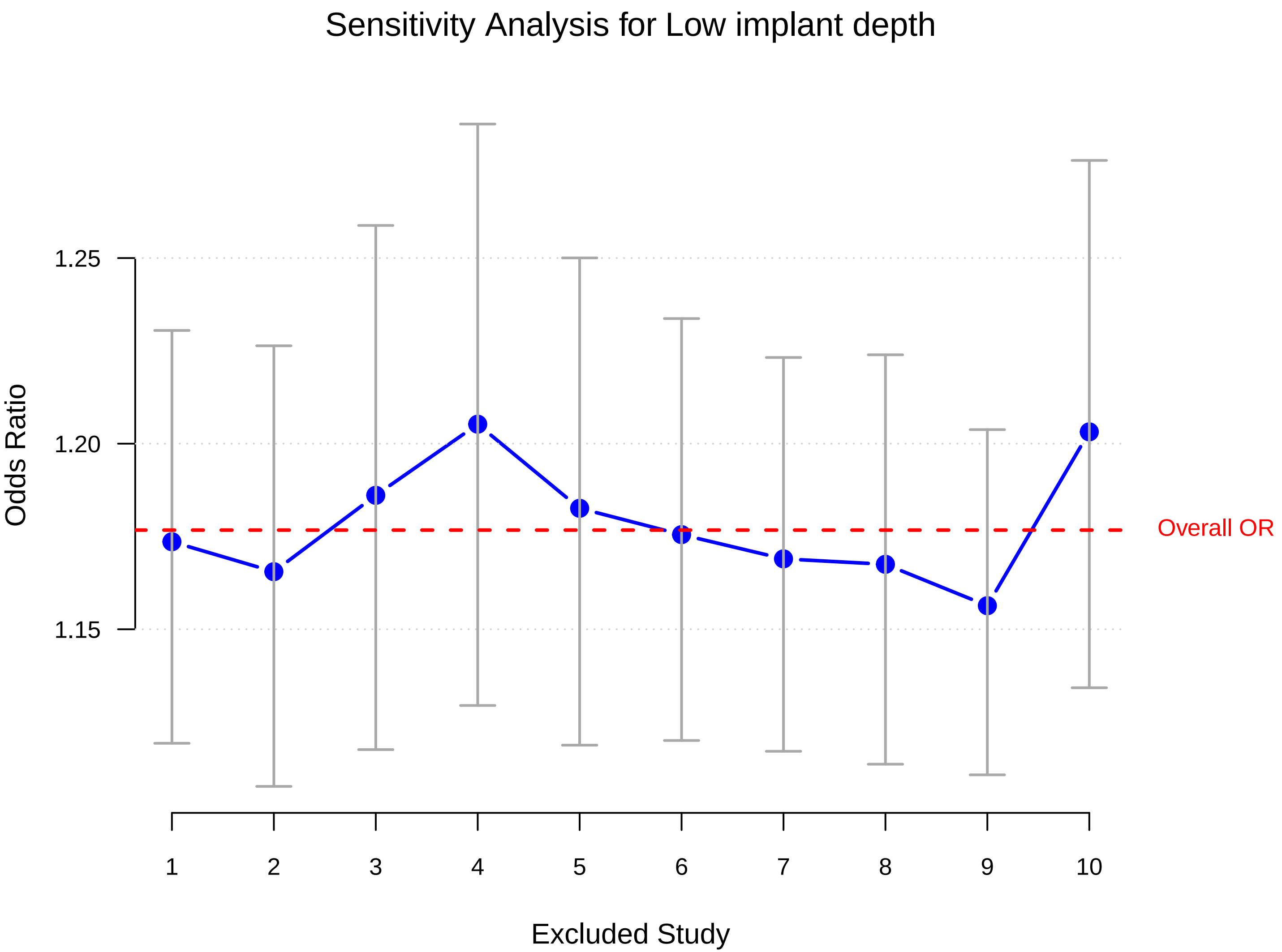

Sensitivity Analysis for Low implant depth
Odds Ratio
1.25
1.20
1.15
Overall OR
1
2
3
4
5
6
7
8
9
10
Excluded Study

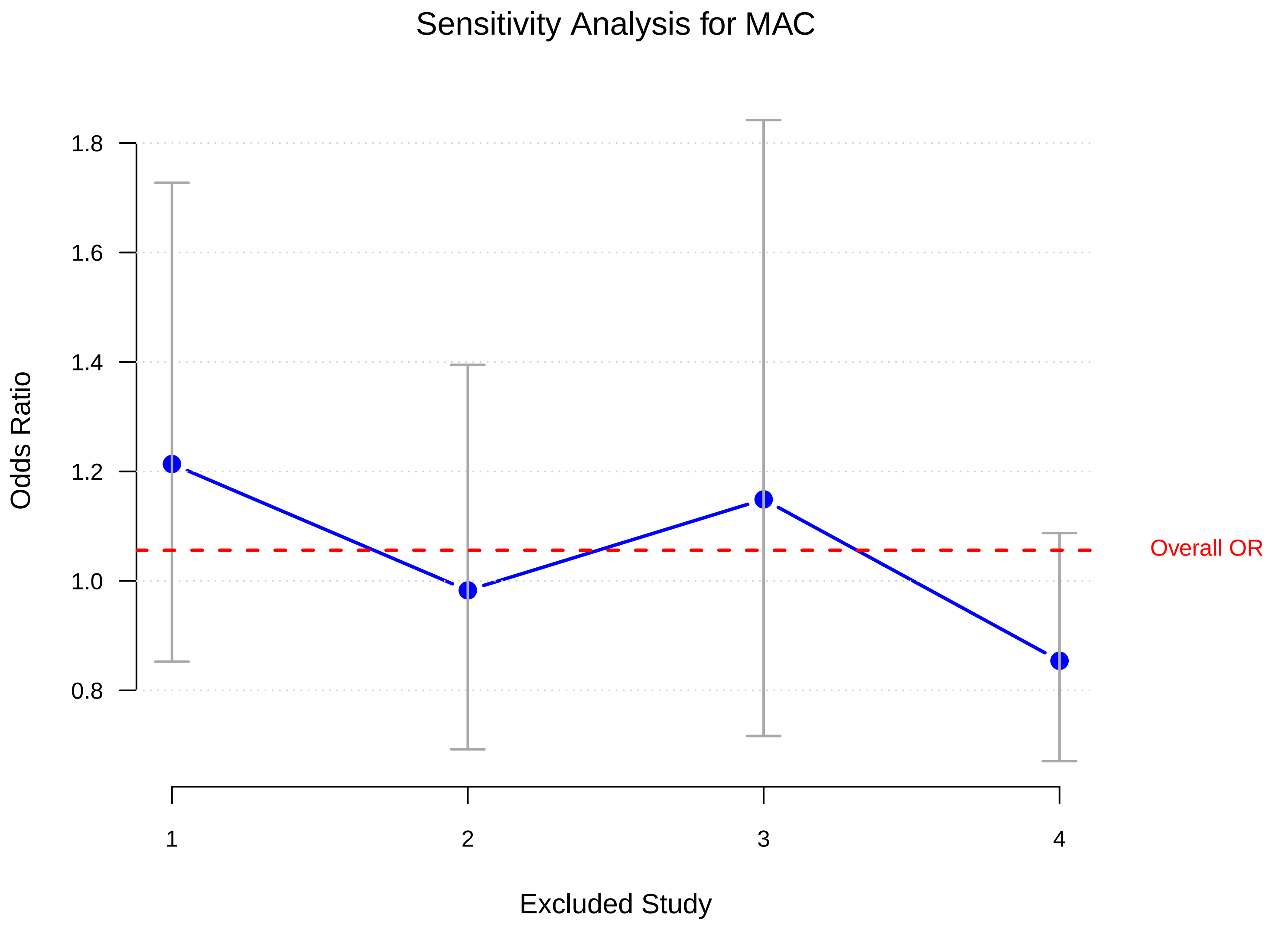

Sensitivity Analysis for MAC
Odds Ratio
1.8
1.6
1.4
1.2
1.0
0.8
Overall OR
1
2
3
4
Excluded Study

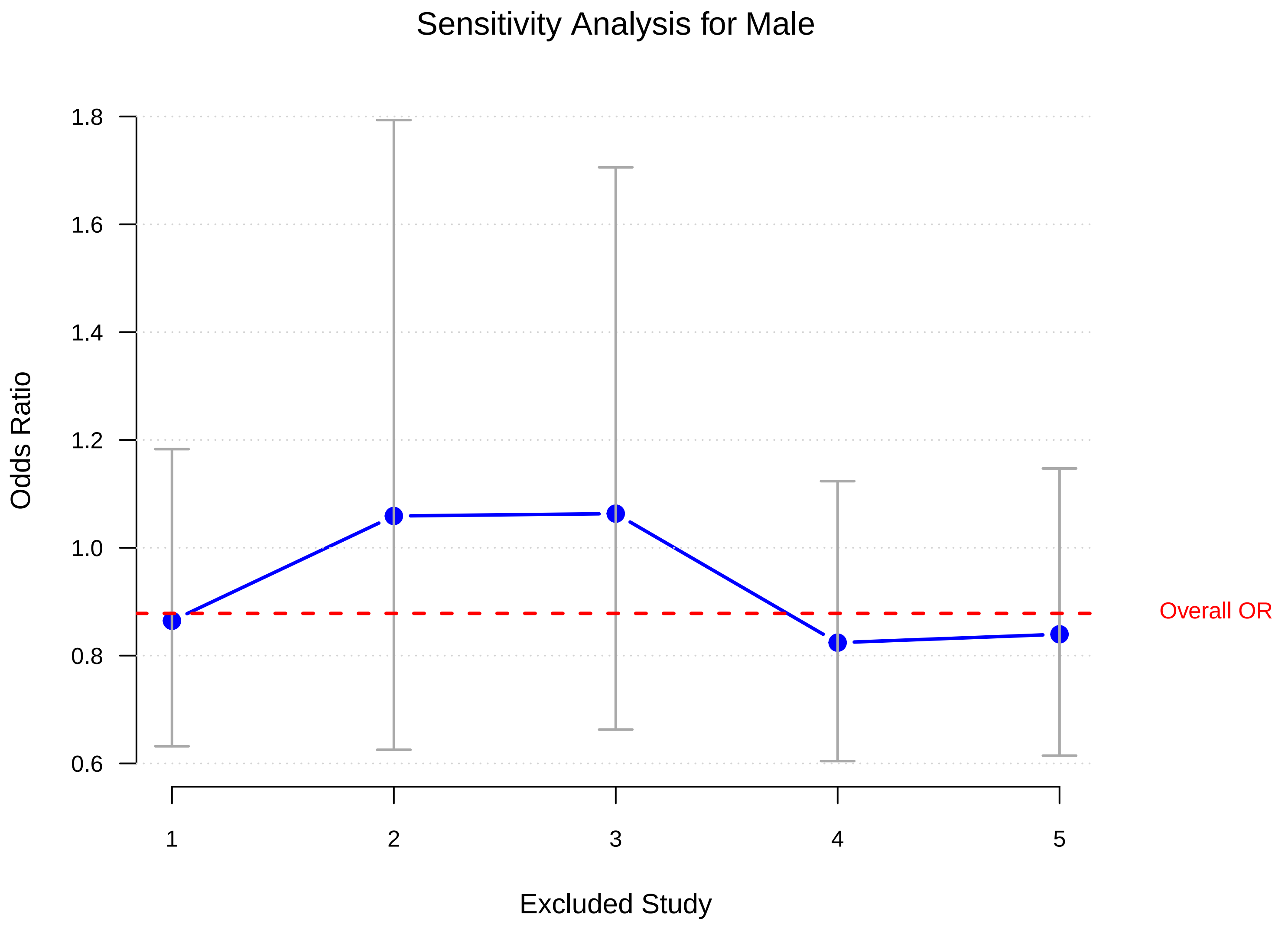

Sensitivity Analysis for Male
Odds Ratio
1.8
1.6
1.4
1.2
1.0
0.8
0.6
Overall OR
1
2
3
4
5
Excluded Study

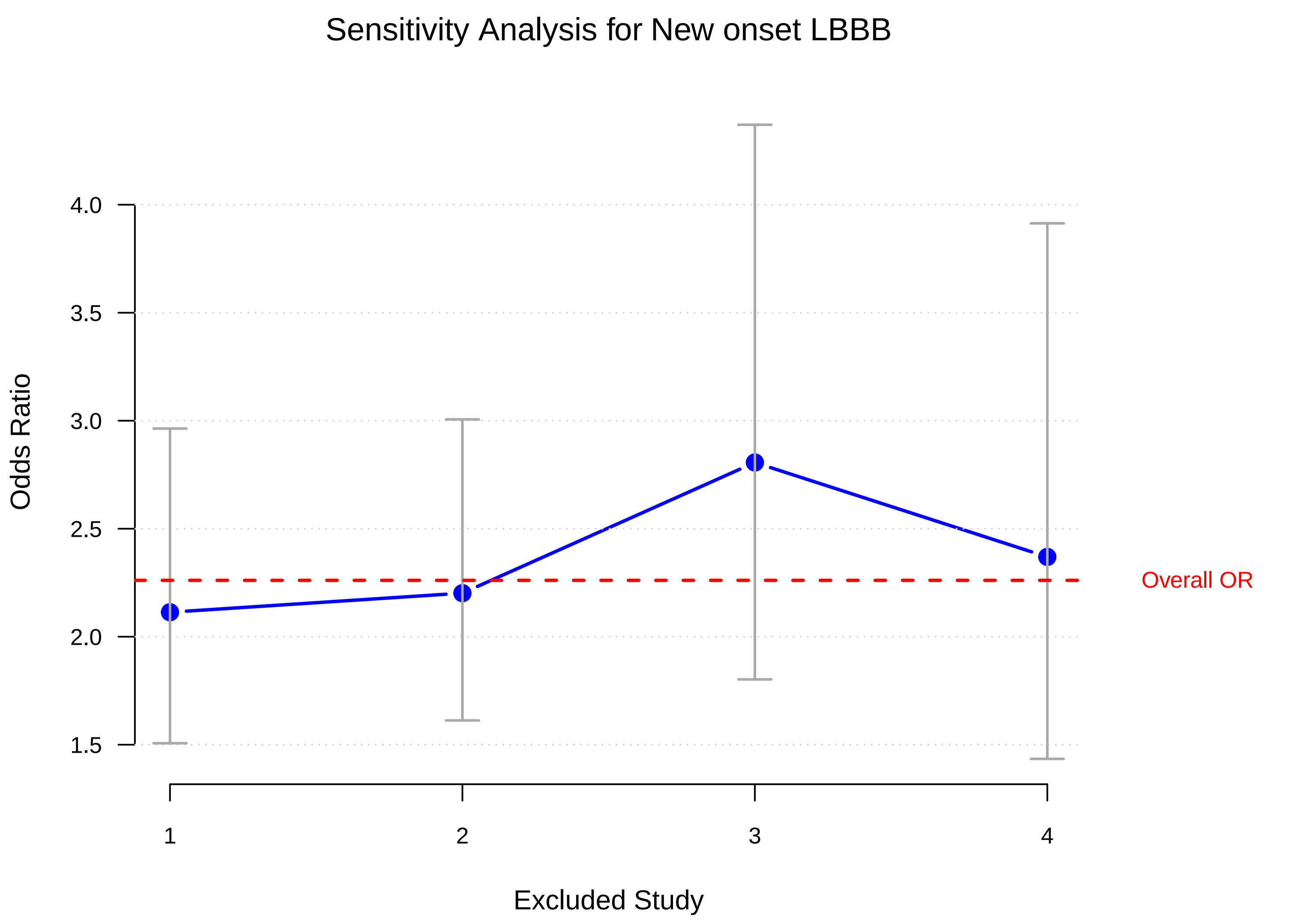

Sensitivity Analysis for New onset LBBB
Odds Ratio
4.0
3.5
3.0
2.5
2.0
1.5
Overall OR
1
2
3
4
Excluded Study

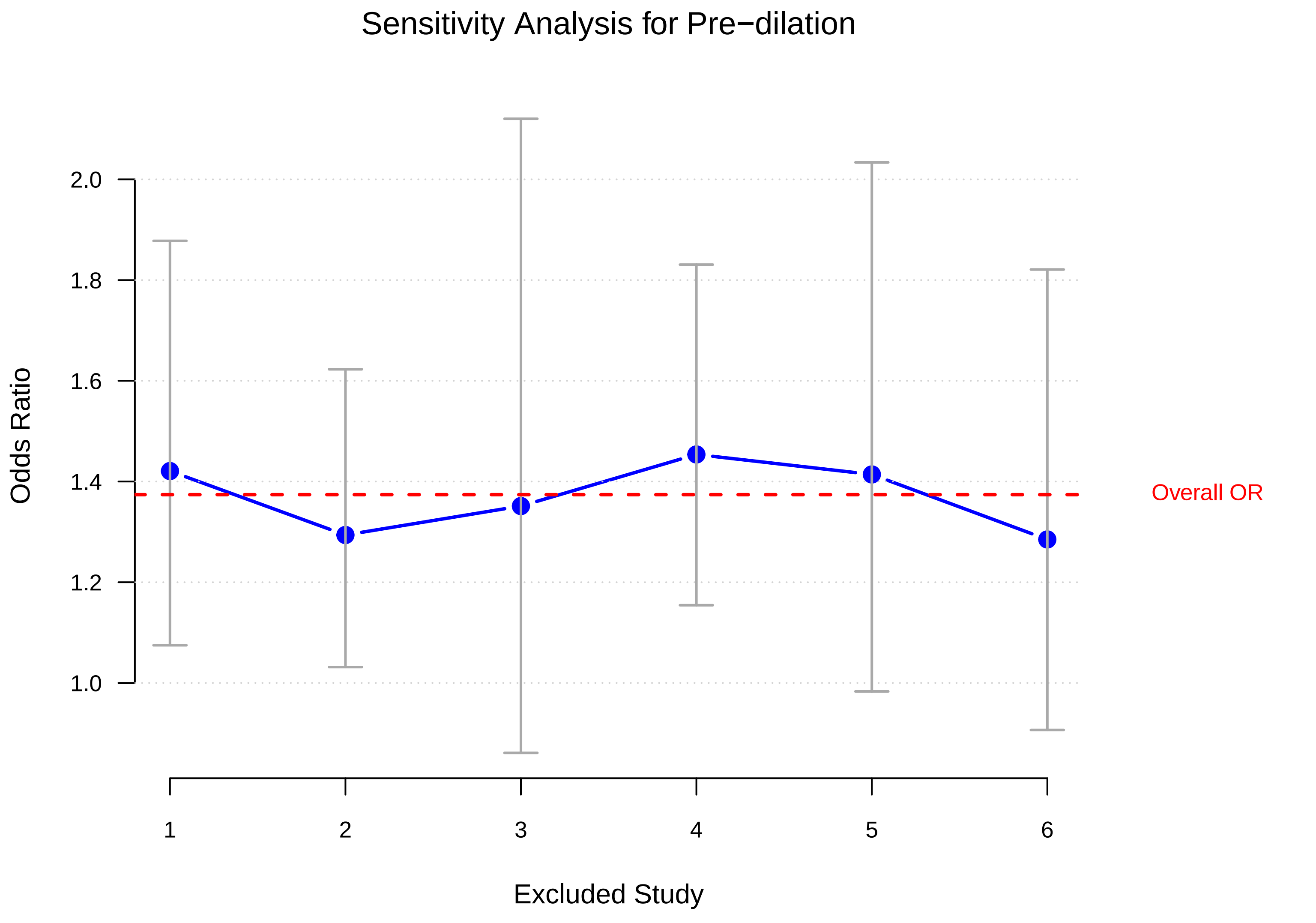

Sensitivity Analysis for Pre−dilation
Odds Ratio
2.0
1.8
1.6
1.4
1.2
1.0
Overall OR
1
2
3
4
5
6
Excluded Study

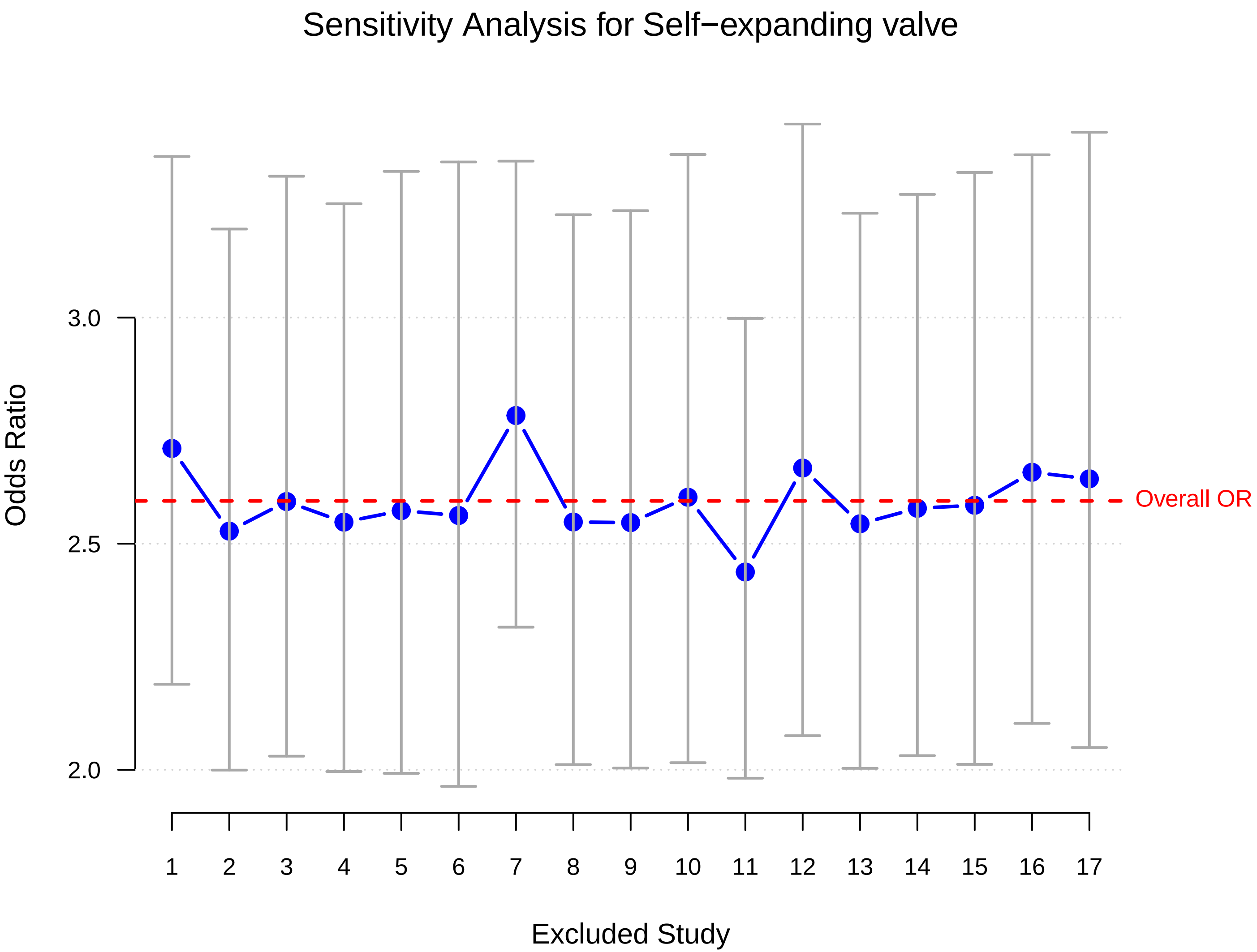

Sensitivity Analysis for Self−expanding valve
Odds Ratio
3.0
2.5
2.0
Overall OR
1
2
3
4
5
6
7
8
9
10
11
12
13
14
15
16
17
Excluded Study

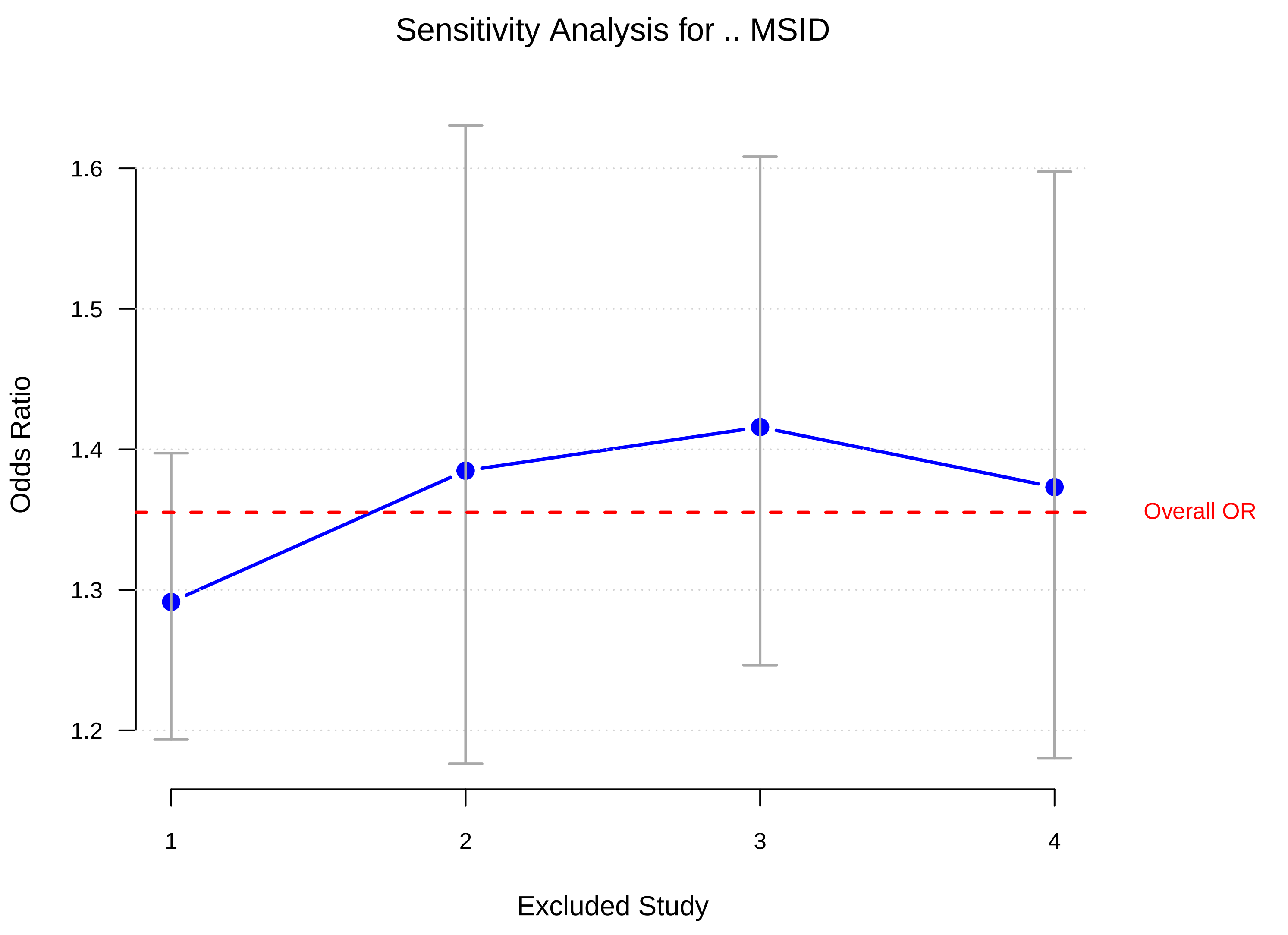

Sensitivity Analysis for .. MSID
Odds Ratio
1.6
1.5
1.4
1.3
1.2
Overall OR
1
2
3
4
Excluded Study
